# Predictors of 30-Day Mortality Among Dutch Patients Undergoing Colorectal Cancer Surgery, 2011-2016

**DOI:** 10.1001/jamanetworkopen.2021.7737

**Published:** 2021-04-26

**Authors:** Tom van den Bosch, Anne-Loes K. Warps, Michael P. M. de Nerée tot Babberich, Christina Stamm, Bart F. Geerts, Louis Vermeulen, Michel W. J. M. Wouters, Jan-Willem T. Dekker, Rob A. E. M. Tollenaar, Pieter J. Tanis, Daniël M. Miedema

**Affiliations:** 1 Laboratory for Experimental Oncology and Radiobiology, Center for Experimental and Molecular Medicine, Cancer Center Amsterdam and Amsterdam Gastroenterology and Metabolism, Amsterdam University Medical Centers, University of Amsterdam, Amsterdam, the Netherlands; 2 Oncode Institute, Utrecht, the Netherlands; 3 Department of Surgery, Leiden University Medical Center, Leiden, the Netherlands; 4 Dutch Institute for Clinical Auditing, Leiden, the Netherlands; 5 PacMed, Amsterdam, the Netherlands; 6 Healthplus.ai, Amsterdam, the Netherlands; 7 Department of Surgical Oncology, Antoni van Leeuwenhoek Hospital, Amsterdam, the Netherlands; 8 Department of Biomedical Data Sciences, Leiden University Medical Center, Leiden, the Netherlands; 9 Department of Surgery, Reinier de Graaf Groep, Delft, the Netherlands; 10 Amsterdam University Medical Centers, Department of Surgery, University of Amsterdam, Cancer Center Amsterdam, Amsterdam, the Netherlands

## Abstract

**Importance:**

Quality improvement programs for colorectal cancer surgery have been introduced with benchmarking based on quality indicators, such as mortality. Detailed (pre)operative characteristics may offer relevant information for proper case-mix correction.

**Objective:**

To investigate the added value of machine learning to predict quality indicators for colorectal cancer surgery and identify previously unrecognized predictors of 30-day mortality based on a large, nationwide colorectal cancer registry that collected extensive data on comorbidities.

**Design, Setting, and Participants:**

All patients who underwent resection for primary colorectal cancer registered in the Dutch ColoRectal Audit between January 1, 2011, and December 31, 2016, were included. Multiple machine learning models (multivariable logistic regression, elastic net regression, support vector machine, random forest, and gradient boosting) were made to predict quality indicators. Model performance was compared with conventionally used scores. Risk factors were identified by logistic regression analyses and Shapley additive explanations (ie, SHAP values). Statistical analysis was performed between March 1 and September 30, 2020.

**Main Outcomes and Measures:**

The primary outcome of this cohort study was 30-day mortality. Prediction models were trained on a training set by performing 5-fold cross-validation, and outcomes were measured by the area under the receiver operating characteristic curve on the test set. Machine learning was further used to identify risk factors, measured by odds ratios and SHAP values.

**Results:**

This cohort study included 62 501 records, most patients were male (35 116 [56.2%]), were aged 61 to 80 years (41 560 [66.5%]), and had an American Society of Anesthesiology score of II (35 679 [57.1%]). A 30-day mortality rate of 2.7% (n = 1693) was found. The area under the curve of the best machine learning model for 30-day mortality (0.82; 95% CI, 0.79-0.85) was significantly higher than the American Society of Anesthesiology score (0.74; 95% CI, 0.71-0.77; *P* < .001), Charlson Comorbidity Index (0.66; 95% CI, 0.63-0.70; *P* < .001), and preoperative score to predict postoperative mortality (0.73; 95% CI, 0.70-0.77; *P* < .001). Hypertension, myocardial infarction, chronic obstructive pulmonary disease, and asthma were comorbidities with a high risk for increased mortality. Machine learning identified specific risk factors for a complicated course, intensive care unit admission, prolonged hospital stay, and readmission. Laparoscopic surgery was associated with a decreased risk for all adverse outcomes.

**Conclusions and Relevance:**

This study found that machine learning methods outperformed conventional scores to predict 30-day mortality after colorectal cancer surgery, identified specific patient groups at risk for adverse outcomes, and provided directions to optimize benchmarking in clinical audits.

## Key Points

**Question:**

Can big-data analysis of clinical audits help to find new risk factors and predict adverse events associated with colorectal cancer surgery?

**Findings:**

This cohort study found that machine learning applied to a clinical audit containing 62 501 records and 103 preoperative variables of surgically treated patients with colorectal cancer outperformed conventional scores in predicting 30-day postoperative mortality but with similar performance as a preexisting case-mix model. New risk factors for several other adverse events may be identified.

**Meaning:**

This study suggests that machine learning methods may be of additional value in analyzing quality indicators in colorectal cancer surgery, thereby providing directions to optimize case-mix corrections for benchmarking in clinical auditing.

## Teaser

This cohort study investigates the ability of machine learning to predict quality indicators for colorectal cancer surgery and identify previously unrecognized predictors of 30-day mortality based on a large nationwide colorectal cancer registry that collected extensive data on comorbidities.

## Introduction

Resection of colorectal cancer is a frequently performed surgical procedure with a generally reported incidence of complications of more than 30%,^1^ and these complications are associated with patient burden and increased health care use.^2,3^ A European study showed that nearly one-third of hospital budgets are spent on treating complications of colorectal cancer surgery.^4^ Identifying patients at risk for complications is thus of significant importance. Previously identified risk factors for postoperative complications are advanced age, higher American Society of Anesthesiology (ASA) score, emergency surgery, comorbidities, and advanced tumor stage.^1,5,6,7,8,9,10,11^

Worldwide, several nationwide registries have been set up to improve health care for specific diseases.^12,13,14^ Providing feedback on hospital performance is a key principle of clinical auditing. However, patient populations might differ between hospitals, which requires case-mix correction for reliable benchmarking.^15,16,17^ Detailed registration of patient characteristics is essential for optimizing case-mix correction but increases registration burden.

The Dutch ColoRectal Audit (DCRA) was initiated in 2009 to improve the quality of surgical care for patients in the Netherlands with colorectal cancer. Nationwide coverage of the DCRA is more than 95%, with high validity of the data.^18^ This audit differs from other registries in the large number of captured comorbidities. Previous studies on the DCRA data mainly used common predictors, such as the ASA score and Charlson Comorbidity Index (CCI),^15,18,19^ leaving the added value of extensive registration of comorbidities largely unexplored.

Machine learning (ML) tools can be used to interrogate large clinical data sets with the goal of improving patient care. For colorectal cancer surgery, ML algorithms have been published for the prediction of postoperative complications with C statistics (a measure of concordance between model-based risk estimates and observed events) ranging from 0.65 to 0.98.^20,21^ Machine learning has further been used to diagnose early-stage colorectal cancer,^22^ predict the waiting time for colorectal cancer surgery,^23^ and predict the prognosis of patients with colorectal cancer.^24^ The aim of this study was to make prediction models for quality indicators, including 30-day mortality, and to identify potentially unrecognized relevant predictors for outcomes after surgery for primary colorectal cancer using ML methods on the extensive DCRA data set.

## Methods

### Study Population

Data of all patients undergoing colorectal cancer surgery between January 1, 2011, and December 31, 2016, were extracted from the DCRA. Patients who received a watch-and-wait strategy without subsequent surgical treatment were excluded. Informed consent for data collection and ethical approval were not required according to Dutch law.^25,26^ This cohort study follows the Strengthening the Reporting of Observational Studies in Epidemiology (STROBE) reporting guideline for observational studies.^27^

### Outcome Parameters

The primary outcome was 30-day mortality. Secondary outcomes included a complicated course (complication resulting in a hospital stay of >14 days, surgical complication requiring a reintervention, or death within 30 days after surgery or while in the hospital), intensive care unit (ICU) admission, prolonged length of stay (LOS; >21 days), and readmission within 30 days.

### Predictive Variables

All 103 available patient, tumor, and preoperatively known variables in the DCRA data set were considered potential predictors (eTable 1 in the Supplement). For the prediction of LOS of more than 21 days and readmission, 14 intraoperative predictors were added (ie, intraoperative complications, additional resections, and laparoscopic conversion).

### Statistical Analysis

Statistical analyses were performed between March 1 and September 30, 2020. Trivial imputation was performed for DCRA case-mix variables (unlikely to be underreported by hospitals), for specific comorbidities if their overlapping variable was set to “none” or if another comorbidity in the same group was registered, and for logically deducible values by combining variables (M stage, tumor location, and conversion) or based on date of introduction (screening). For nontrivial missing data, values were assumed to be missing at random, and the k-nearest neighbor imputation with N = 3 was used, which has been shown to introduce minimal bias compared with using complete observations.^28^ Continuous variables were converted to categorical variables as is done in standard DCRA case mixing: age (<60, 60-69, 70-79, and ≥80 years) and body mass index (calculated as weight in kilograms divided by height in meters squared; <18.5, 18.5-25, >25-30, and >30).

Prediction models were created by splitting the data set chronologically into a test set containing 19% of the patients (2016) and a training set containing 81% (2011-2015). Logistic regression (LR), elastic net regression,^29^ random forest,^30^ and gradient boosting method^31^ models were trained by performing 5-fold cross-validation on the training set using stratified splitting in equally sized groups. For mortality, a support vector machine^32,33^ model was also trained. For all models, different methods of handling data were tested: balancing of response variables and adding of missing flags for data that were imputed, creating 4 possible models for each method and response (support vector machine was trained only with balancing). The predictive strength of the models was measured by the area under the receiver operating characteristic curve (AUC) on the test set after performing hyperparameter training on the training set. Pairwise comparisons of AUCs were performed by the test of DeLong et al.^34^

The impact of risk factors was predicted by odds ratios (ORs) or regression coefficients (β) with 95% CIs. *P* values (2-sided Wald test) were reported for the unbalanced LR model without missing flags of each outcome parameter. No prior significance was assumed. Model assumptions were checked using variance inflation factors to measure collinearity among variables,^35,36^ with variance inflation factors greater than 5 used as a cutoff for potential multicollinearity.^37^ Likelihood ratio tests and the Akaike Information Criterion were used to validate the use of the full variable set by comparing against nested models.

Shapley additive explanations (ie, SHAP values)^38^ were calculated for the unbalanced gradient boosting method model without missing flags to further analyze the association of patient characteristics. SHAP values quantify the association of a variable with the outcome of a single patient, and the mean absolute SHAP value across all patients is reported as the SHAP value of a variable.

For mortality, AUCs of ML models were compared with the AUC of the preoperative score to predict postoperative mortality (POSPOM), CCI, and ASA score. The AUCs were also compared with LR applied to the DCRA case-mix data set. The DCRA case-mix data set contains the currently used DCRA case-mix variables: sex, body mass index, age, CCI, ASA score, preoperative tumor complications, urgency of the resection, additional resection due to metastasis or tumor ingrowth, T stage, and M stage.

All analyses were performed using R, version 3.6.1 and RStudiod version 1.2.1335 software (R Project for Statistical Computing). Pipelining and data splitting were performed using the Caret package, version 6.0-86, in RStudio. Modeling was performed using the randomForest, version 4.6-14; xgboost, version 1.2.0.1; and kernlab, version 0.9-29 packages in RStudio. Receiver operating characteristic curves and AUC scores were generated using the pROC, version 1.16.2 package in RStudio. The SHAP values were calculated using the SHAPforxgboost, version 0.0.4 package in RStudio. *P* < .05 was considered significant.

## Results

### Study Population

**Figure 1.**
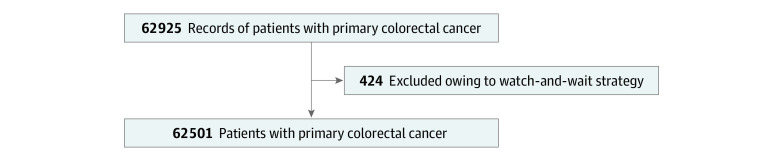
Patient Inclusion Criteria

A total of 62 925 records of patients with primary colorectal cancer were included in the DCRA between January 1, 2011, and December 31, 2016. After excluding 424 records of patients who followed a watch-and-wait strategy, 62 501 records of 62 151 (99.4%) unique, surgically treated patients were included in the final data set (Figure 1). A total of 0.6% of values were missing among the 103 variables (0.65 missing per patient; 46 474 [74.4%] complete cases), and 0.7% of values were missing among the 117 variables in the preoperative data set (0.80 missing per patient; 43 588 [69.7%] complete cases). The chronologically split training set and test set are shown in eTable 1 in the Supplement.

The overall study population consisted predominantly of male patients (35 116 [56.2%]) (eTable 1 in the Supplement). Most patients were aged between 61 and 80 years (41 460 [66.5%]) and had an ASA score of II (35 679 [57.1%]). The most common comorbidities were hypertension (36.1%), type 2 diabetes (11.6%), chronic obstructive pulmonary disease (COPD) and asthma (10.9%), atrial fibrillation or flutter (8.3%), and a history of myocardial infarction (6.2%) (eTable 1 in the Supplement).

A total of 21 748 patients (34.8%) presented with tumor-related complications, mostly obstruction (10.9%) or blood loss or anemia (17.2%). Most patients underwent a laparoscopic resection (59.1%), which was converted to open surgery for 7.1% of patients.

A total of 20 363 (32.6%) patients experienced at least 1 complication, and 1693 patients (2.7%) died within 30 days after surgery. A total of 11 443 patients (18.3%) fulfilled the criteria of a complicated course, 11 931 (19.1%) were admitted to the ICU, 4874 (7.8%) had an LOS greater than 21 days, and 4496 (7.2%) were readmitted.

### 30-Day Mortality

**Figure 2.**
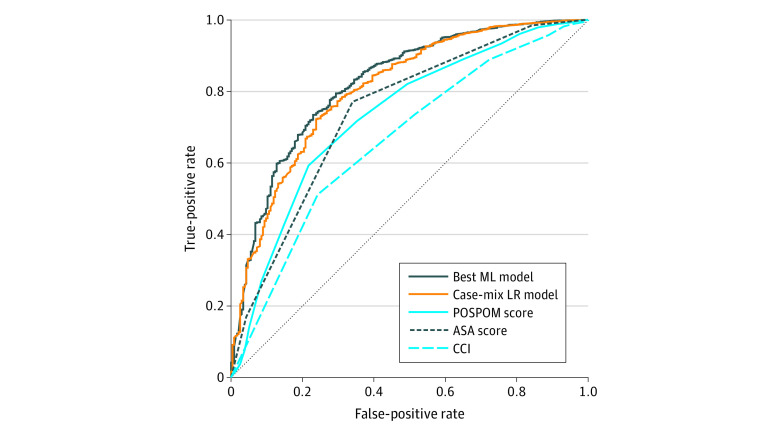
Receiver Operating Characteristic Plot for 30-Day Mortality Accuracy of 30-day mortality prediction for the best performing machine learning (ML) model (elastic net regression), case-mix logistic regression (LR) model, the preoperative score to predict postoperative mortality (POSPOM), American Society of Anesthesiology (ASA) score, and Charlson Comorbidity Index (CCI).

The AUC of the best ML model (elastic net regression) for 30-day mortality was 0.82 (95% CI, 0.79-0.85), with no significantly different AUCs among the best ML models (eFigure 1 and eTable 2 in the Supplement). The best ML model performed significantly better than the ASA score (AUC, 0.74; 95% CI, 0.71-0.77; *P* < .001), POSPOM (AUC, 0.73; 95% CI, 0.70-0.77; *P* < .001), CCI (AUC, 0.66; 95% CI, 0.63-0.70; *P* < .001), and DCRA case-mix regression model (AUC, 0.81; 95% CI, 0.78-0.84; *P* = .01) (Table^34^ and Figure 2 and eTable 2 in the Supplement).

**Table.** AUC Scores for All Outcome Measures

Outcome measure	Best machine learning model	DCRA case-mix regression model	ASA score	POSPOM	CCI
Mortality	0.82 (0.79-0.85)	0.81 (0.78-0.84)	0.74 (0.71-0.77)	0.73 (0.70-0.77)	0.66 (0.63-0.70)
*P* value^a^	NA	.01	1.1 × 10^−10^	1.4 × 10^−10^	6.0 × 10^−17^
Complicated course	0.68 (0.67-0.69)	NA	NA	NA	NA
Prolonged length of stay	0.71 (0.69-0.73)	NA	NA	NA	NA
Readmission	0.63 (0.61-0.65)	NA	NA	NA	NA
ICU admission	0.74 (0.72-0.75)	NA	NA	NA	NA

Abbreviations: ASA, American Society of Anesthesiology; AUC, area under the receiver operating characteristic curve; CCI, Charlson Comorbidity Index; DCRA, Dutch ColoRectal Audit; ICU, intensive care unit; NA, not applicable; POSPOM, preoperative score to predict postoperative mortality.

^a^ Reported *P* values are in comparison with the best machine learning model and were calculated using the test of DeLong et al.^34^

Multicollinearity (variance inflation factor >5) was found in levels of 6 (0.4%) categorical variables, which indicates low multicollinearity among different variables (eTable 3 in the Supplement) and validates the regression assumptions. The goodness of fit was better for the LR model than for nested models, as shown by a lower Akaike Information Criterion of the LR model than of nested models and by the likelihood ratio test (eTable 4 in the Supplement). This goodness-of-fit analysis showed that feature selection before risk analysis to avoid overfitting is not necessary.

**Figure 3.**
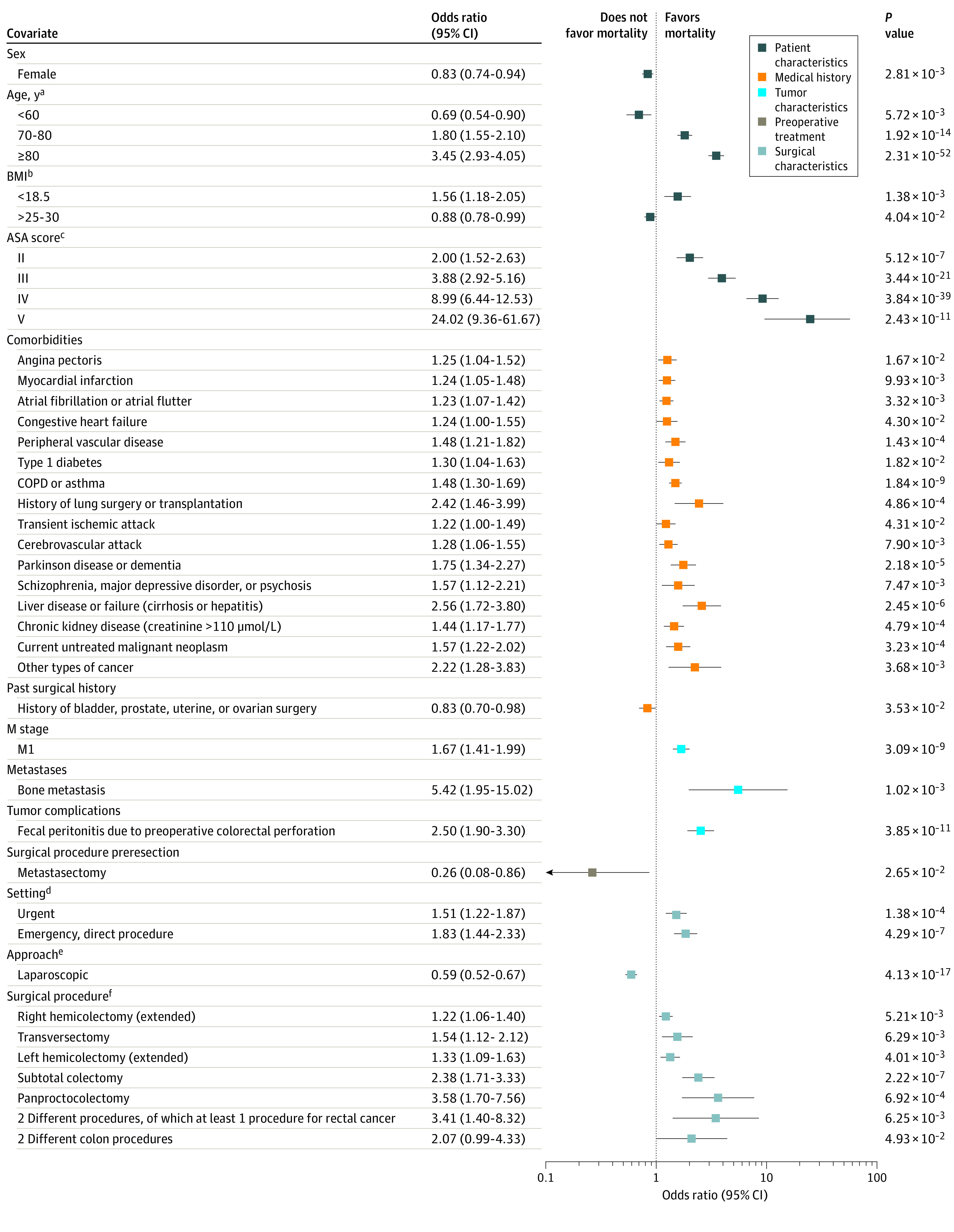
Significant Predictors of 30-Day Mortality Logistic regression model of 30-day mortality for 62 501 patients. All regression coefficients with *P* < .05 are translated to odds ratios. For categorical variables, references are shown on the right axis. To convert creatinine to mg/dL, divide by 88.4. BMI indicates body mass index (calculated as weight in kilograms divide by height in meters squared); COPD, chronic obstructive pulmonary disease. ^a^Reference, 60-70 years. ^b^Reference, 18.5-25. ^c^Reference, American Society of Anesthesiology (ASA) score I. ^d^Reference, elective setting. ^e^Reference, open approach. ^f^Reference, low anterior resection or sigmoid resection.

The ORs of all significant variables for 30-day mortality are shown in Figure 3, and all regression coefficients are shown in eTable 5 in the Supplement. Patient characteristics with the highest increase in risk were being older than 80 years (OR, 3.45; 95% CI, 2.93-4.05; *P* < .001), body mass index less than 18.5 (OR, 1.56; 95% CI, 1.18-2.05; *P* < .001), and ASA scores of III (OR, 3.88; 95% CI, 2.92-5.16; *P* < .001), IV (OR, 8.99; 95% CI, 6.44-12.53; *P* < .001), and V (OR, 24.02; 95% CI, 9.36-61.67; *P* < .001). The comorbidities with the highest significant risks for 30-day mortality were liver failure (OR, 2.56; 95% CI, 1.72-3.80; *P* < .001), medical history of lung surgery or transplant (OR, 2.42; 95% CI, 1.46-3.99; *P* < .001), and history of other types of cancer (OR, 2.22; 95% CI, 1.28-3.83; *P* = .004). Fecal peritonitis (OR, 2.50; 95% CI, 1.90-3.30; *P* < .001) and bone metastasis (OR, 5.42; 95% CI, 1.95-15.02; *P* = .001) at presentation also increased the risk of mortality. Surgical procedures associated with high risk were panproctocolectomy (OR, 3.58; 95% CI, 1.70-7.56; *P* < .001)*,* resection for multiple tumors with at least 1 rectal procedure (OR, 3.41; 95% CI, 1.40-8.32; *P* = .001), and subtotal colectomy (OR, 2.38; 95% CI, 1.71-3.33; *P* < .001).

**Figure 4.**
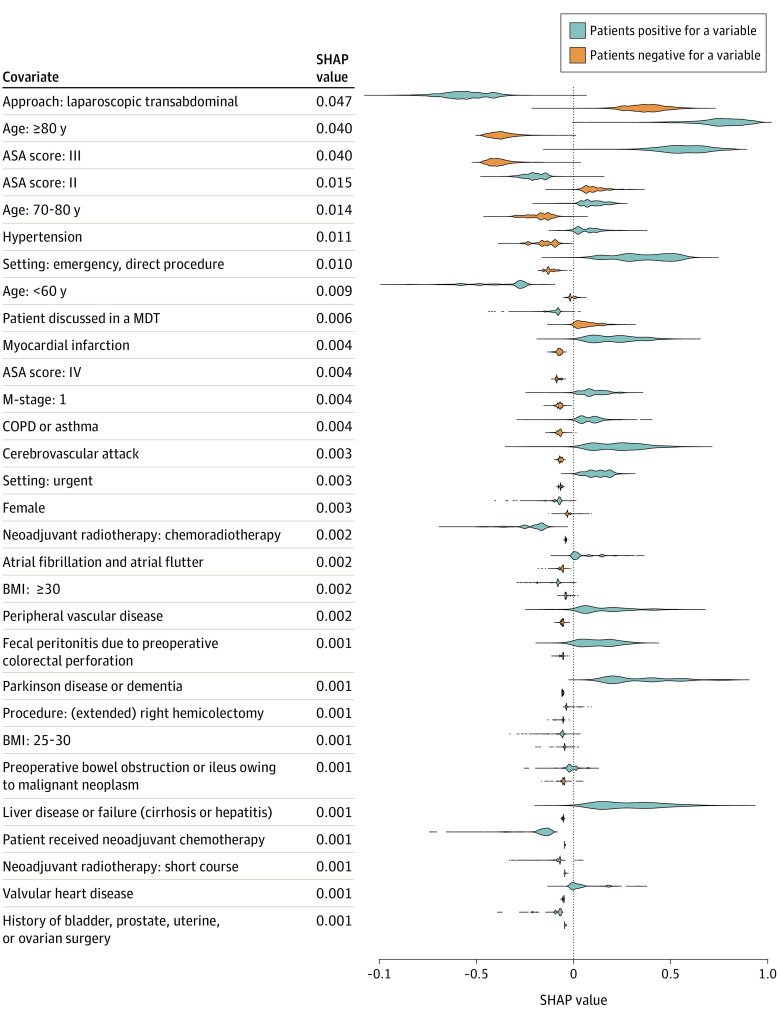
Variables That Demonstrated the Greatest Association With Prediction of 30-Day Mortality Top 30 Shapley additive explanation (SHAP) feature values of the gradient-boosting model for prediction of 30-day mortality. SHAP values were calculated per variable for all patients in the test set. Distributions of SHAP values for patients are shown in blue (patients who are positive for a variable) and orange (patients who are negative for a variable). SHAP values were ranked by the mean of the absolute value across all patients in the test set. ASA indicates American Society of Anesthesiology; BMI, body mass index (calculated as weight in kilograms divided by height in meters squared); COPD, chronic obstructive pulmonary disease; and MDT, multidisciplinary team.

Figure 4 shows the top 30 variables with the highest SHAP values of the gradient boosting method model for 30-day mortality. The most important factors associated with the predictive power of the model were age, ASA score, and laparoscopic surgery. The comorbidities with the most predictive power were hypertension, myocardial infarction, and COPD and asthma.

### Secondary Outcomes

The highest AUCs of ML models for secondary outcomes were 0.68 (95% CI, 0.67-0.69) for complicated course, 0.74 (95% CI, 0.72-0.75) for ICU admission, 0.71 (95% CI, 0.69-0.73) for LOS greater than 21 days, and 0.63 (95% CI, 0.61-0.65) for readmission. Overall, the random forest model performed significantly worse for predicting complicated course and ICU admission compared with LR and elastic net regression (eTable 2 and eFigure 1 in the Supplement). There was no best method for data handling because balancing of the data significantly increased the AUC for 12 models and decreased the AUC for 6 models, and adding missing flags significantly increased the AUC for 6 models and decreased the AUC for 2 models.

The regression coefficients for complicated course, ICU admission, LOS greater than 21 days, and readmission are shown in eFigures 2 to 5 and eTables 6 to 9 in the Supplement. Comorbidities associated with an increased risk of a complicated course were pulmonary fibrosis (OR, 1.84; 95% CI, 1.14-2.98; *P* = .01), cardiac valve replacement (OR, 1.43; 95% CI, 1.20-1.71; *P* < .001), and liver disease or failure (OR, 1.46; 95% CI, 1.17-1.82; *P* < .001). Relevant comorbidities for ICU admission were dialysis-dependent kidney failure (OR, 2.27; 95% CI, 1.32-3.91; *P* = .003), history of lung surgery or transplant (OR, 2.01; 95% CI, 1.49-2.71; *P* < .001), and hemiplegia or paraplegia (OR, 1.90; 95% CI, 1.26-2.88; *P* < .001). An increased risk of readmission was found for hypoparathyroidism and hyperparathyroidism (OR, 1.58; 95% CI, 1.01-2.47; *P* = .04), inflammatory bowel disease (OR, 1.34; 95% CI, 1.01-1.79; *P* = .04), and cardiovascular comorbidities (eg, atrial fibrillation or flutter [OR, 1.14; 95% CI, 1.02-1.28; *P* = .02], aortic aneurysm [OR, 1.29; 95% CI, 1.06-1.58; *P* = .001], cerebrovascular attack [OR, 1.22; 95% CI, 1.05-1.41; *P* = .006], and pulmonary embolism [OR, 1.38; 95% CI, 1.09-1.75; *P* = .001]). Laparoscopy was associated with a decreased risk for all postoperative outcomes (complicated course: OR, 0.69; 95% CI, 0.65-0.72; *P* < .001; ICU admission: OR, 0.56; 95% CI, 0.53-0.58; *P* < .001; LOS >21 days: OR, 0.62; 95% CI, 0.57-0.67; *P* < .001) except for readmission (OR, 1.13; 95% CI, 1.05-1.22; *P* = .001). Subtotal colectomy was the surgical procedure associated with the highest increase in risk for complicated course (OR, 2.98; 95% CI, 2.52-3.52; *P* < .001) and ICU admission (OR, 2.02; 95% CI, 1.69-2.41; *P* < .001).

The SHAP values for the secondary outcomes (eFigures 6-9 in the Supplement) showed that age, sex, ASA score, laparoscopy, T4 stage, and emergency surgery were important predictors for complicated course, ICU admission, and LOS greater than 21 days. Rectal cancer, loop ileostomy, and ASA score were important predictors for readmission.

## Discussion

This study explored the added value of applying ML methods to a large, nationwide clinical audit for identifying new risk factors and predicting adverse outcomes after colorectal cancer surgery. Machine learning models based on a colorectal cancer registry including 103 preoperative variables showed better performance (AUC = 0.82) for predicting 30-day mortality than the ASA score, CCI, and POSPOM. Machine learning models for predicting a complicated course, ICU admission, LOS greater than 21 days, and readmission showed AUCs between 0.63 and 0.74. The models provided valuable information on the importance of both well-known risk factors as well as new risk factors for various postoperative outcome parameters for patients undergoing colorectal cancer surgery.

Important predictive information for mortality is lost owing to the limited number of variables in the ASA score (n = 1), CCI (n = 16), and POSPOM score (n = 17). This finding is in line with the results of a systematic review by Goldstein et al,^39^ which demonstrated that predictive models based on electronic healthcare records use a median of 27 variables. Although not based on electronic healthcare records, the case-mix regression model of the DCRA uses 26 variables (including 16 variables in the CCI). The predictive value of the best ML model with more than 100 variables was only slightly better compared with the DCRA case-mix regression model using 26 variables, although the difference was statistically significant. Moreover, the assessment of different methods of data handling showed no single best method for all outcomes, indicating that the limiting factor for predicting quality indicators is associated with the variables in the data set rather than the methods used.

The LR and SHAP analyses identified several known risk factors,^1,5,6,7,8,9,10,11^ each with varying importance for the different outcomes. The LR analysis reveals some rare but high-impact comorbidities, such as pulmonary fibrosis, lung surgery or transplant, cardiac valve replacement, and liver failure, which are important to consider for clinical decision-making on an individual basis in daily practice. Our SHAP analyses revealed that the ASA score and the specific comorbidities of COPD and asthma, hypertension, and myocardial infarction are important variables for predicting postoperative mortality.

Notably, the risk for 30-day mortality is increased most by liver failure in the LR model, followed by a medical history of lung surgery or transplant. However, the SHAP analysis showed that COPD and asthma have the highest predictive value for 30-day mortality and that liver disease had a much lower predictive value. The discrepancy between regression coefficients and SHAP values can be explained by the prevalence of a variable. Odds ratios are calculated only for patients with whom that variable is associated, whereas mean SHAP values are calculated across all patients. Hence, a variable with low impact and high prevalence will have a low OR but a high SHAP value.

Laparoscopic surgery showed a decreased risk and a high predictive value for 30-day mortality, complicated course, ICU admission, and LOS greater than 21 days. Nevertheless, this parameter should probably not be used as a case-mix factor because there might be specific reasons to still perform open surgery. However, this finding stresses the importance of implementing minimally invasive surgery for colorectal cancer surgery in any center worldwide. Laparoscopic colorectal surgery was studied in multiple trials (COREAN [Comparison of Open Versus Laparoscopic Surgery for Mid or Low Rectal Cancer After Neoadjuvant Chemoradiotherapy], COLOR [Colon Carcinoma Laparoscopic or Open Resection], CLASICC [Capecitabine and Oxaliplatin Adjuvant Study in Stomach Cancer] trial, ACOSOG [American College of Surgeons Oncology Group] Z6051, and ALaCaRT [Australasian Laparoscopic Cancer of the Rectum])^40,41,42,43,44,45,46,47^ but often failed to demonstrate clear benefits associated with its use. In contrast, large population-based studies demonstrated a lower risk of postoperative mortality and cardiopulmonary complications.^48,49,50,51,52^ The present analyses confirm these findings together with seldom-reported benefits associated with the reduction in LOS greater than 21 days and ICU admission.

For complicated course, our results showed that several specific comorbidities, such as COPD and asthma, atrial fibrillation or flutter, and previous types of cancer, had a high predictive value. Previous studies demonstrated that comorbidities, in general, did not increase the risk of postoperative complications but that COPD and asthma^1,53^ and cardiovascular complications^54^ did increase this risk. In contrast with our results, diabetes decreased the risk of complications,^1^ and neurologic comorbidities were independently associated with complications.^53^ Furthermore, we found that younger age was associated with high risk for readmission. This finding is in line with a study by Berry et al^55^ that evaluated 30-day readmission for 31 729 762 US patients and concluded that younger patients were more likely to be readmitted. Berry et al^55^ suggested that the relatively high readmission rates of young patients can be explained by the competing risk of postdischarge death.

Our results have important implications for clinical audits and case-mix corrections that may be relevant to other registries and countries. First, we found that, for each outcome, different risk factors are important, suggesting that a different set of variables should be used for the case-mix correction for different outcomes. Currently, at least in the DCRA, 1 set of case-mix variables is used for all outcomes. Second, we found a minimal, although significant, increase in predictive value when ML models were applied to the full data set, including 103 variables compared with the reduced data set of 26 variables. Hence, we conclude that the registration burden in audits can be considerably reduced without much loss of predictive value.

### Limitations

This study has some limitations. Errors and changes in data collection, such as incomplete registration and changing registration policies, may have affected both performance and generalizability.^56,57^ Furthermore, biases in data collection undermine the assumption of the values missing at random used for imputation, reducing the strength of imputation methods. Selection bias should be acknowledged as a limiting factor for the prediction models because the decision to perform a resection is made by the surgeon and patient before registration in the DCRA. Given the large number of patients (n = 62 501) and the high data validity and completeness of the DCRA,^18^ further increasing the sample size to improve the models is not likely to be of additional value. Further research could thus better focus on data sets containing more biological information (eg, blood test values, imaging, and genomic data), more “live” patient data (eg, current medication use, blood pressure, and heart rates), or a combination of biological and clinical data. Ultimately, combined longitudinal information could be collected through various systems, such as the electronic healthcare record, genomic databases, and wearable devices.^58^

## Conclusions

Prediction models based on a clinical audit consisting of 103 preoperative variables of 62 501 surgically treated colorectal tumors performed better at predicting 30-day mortality than the POSPOM, ASA score, and CCI as well as the case-mix regression model, but the AUCs of the prediction models are too low for direct clinical implementation. However, we demonstrated that the ML models are able to identify factors associated with postoperative quality of care outcomes. It was found that minimally invasive surgery is associated with increased quality of care as assessed by several outcomes. This study also demonstrated that variables are not equally predictive for all outcomes, suggesting that applying different case-mix models in clinical auditing improves the reliability of benchmarking.

## Notes

Supplement. **eTable 1.** Characteristics of 62 501 Patient Records Included in This Study **eTable 2.** Machine Learning Model Specifications for Each Outcome **eTable 3.** Multicollinearity in the DCRA Dataset **eTable 4.** Goodness-of-Fit of Multivariate Logistic Regression Model **eTable 5.** Multivariate Logistic Regression Analyses for Mortality After Colorectal Cancer Surgery **eTable 6.** Multivariate Logistic Regression Analyses for Complicated Course After Colorectal Cancer Surgery **eTable 7.** Multivariate Logistic Regression Analyses for ICU Admission After Colorectal Cancer Surgery **eTable 8.** Multivariate Logistic Regression Analyses for Readmission After Colorectal Cancer Surgery **eTable 9.** Multivariate Logistic Regression Analyses for Prolonged Stay in Hospital After Colorectal Cancer Surgery **eFigure 1.** Pairwise AUC Comparisons for All Models **eFigure 2.** Significant Predictors in Multivariate Logistic Regression Models for Complicated Course **eFigure 3.** Significant Predictors in Multivariate Logistic Regression Models for ICU Admission **eFigure 4.** Significant Predictors in Multivariate Logistic Regression Models for Readmission **eFigure 5.** Significant Predictors in Multivariate Logistic Regression Models for Prolonged Length of Hospital Stay **eFigure 6.** Most Influential Predictor Variables for Complicated Course **eFigure 7.** Most Influential Predictor Variables for ICU Admission **eFigure 8.** Most Influential Predictor Variables for Hospital Readmission **eFigure 9.** Most Influential Predictor Variables for Prolonged Length of Hospital Stay
