# Error in Byline

**DOI:** 10.1001/jamanetworkopen.2021.27694

**Published:** 2021-08-19

In the Original Investigation titled “Predictors of 30-Day Mortality Among Dutch Patients Undergoing Colorectal Cancer Surgery, 2011-2016,”^1^ published April 26, 2021, the name of the eighth author should read “Jan Willem T. Dekker,” not “Jan-Willem T. Dekker.” This article has been corrected.
