# Supplementary material for: Predictors of 30-Day Mortality Among Dutch Patients Undergoing Colorectal Cancer Surgery, 2011-2016
Source: JAMA Netw Open. 2021 Apr 26;4(4):e217737. doi: 10.1001/jamanetworkopen.2021.7737 (PMC8076964; doi:10.1001/jamanetworkopen.2021.7737)
Supplement: Supplement. — eTable 1. Characteristics of 62 501 Patient Records Included in This Study eTable 2. Machine Learning Model Specifications for Each Outcome eTable 3. Multicollinearity in the DCRA Dataset eTable 4. Goodness-of-Fit of Multivariate Logistic Regression Model eTable 5. Multivariate Logistic Regression Analyses for Mortality After Colorectal Cancer Surgery eTable 6. Multivariate Logistic Regression Analyses for Complicated Course After Colorectal Cancer Surgery eTable 7. Multivariate Logistic Regression Analyses for ICU Admission After Colorectal Cancer Surgery eTable 8. Multivariate Logistic Regression Analyses for Readmission After Colorectal Cancer Surgery eTable 9. Multivariate Logistic Regression Analyses for Prolonged Stay in Hospital After Colorectal Cancer Surgery eFigure 1. Pairwise AUC Comparisons for All Models eFigure 2. Significant Predictors in Multivariate Logistic Regression Models for Complicated Course eFigure 3. Significant Predictors in Multivariate Logistic Regression Models for ICU Admission eFigure 4. Significant Predictors in Multivariate Logistic Regression Models for Readmission eFigure 5. Significant Predictors in Multivariate Logistic Regression Models for Prolonged Length of Hospital Stay eFigure 6. Most Influential Predictor Variables for Complicated Course eFigure 7. Most Influential Predictor Variables for ICU Admission eFigure 8. Most Influential Predictor Variables for Hospital Readmission eFigure 9. Most Influential Predictor Variables for Prolonged Length of Hospital Stay [file jamanetwopen-e217737-s001.pdf]

# Supplementary Online Content

van den Bosch T, Warps ALK, de Nerée tot Babberich MPM, et al; Dutch ColoRectal Audit. Predictors of 30-day mortality among Dutch patients undergoing colorectal cancer surgery, 2011-2016. *JAMA Netw Open.* 2021;4(4):e217737. doi:10.1001/jamanetworkopen.2021.7737

**eTable 1.** Characteristics of 62 501 Patient Records Included in This Study
**eTable 2.** Machine Learning Model Specifications for Each Outcome
**eTable 3.** Multicollinearity in the DCRA Dataset
**eTable 4.** Goodness-of-Fit of Multivariate Logistic Regression Model
**eTable 5.** Multivariate Logistic Regression Analyses for Mortality After Colorectal Cancer Surgery
**eTable 6.** Multivariate Logistic Regression Analyses for Complicated Course After Colorectal Cancer Surgery
**eTable 7.** Multivariate Logistic Regression Analyses for ICU Admission After Colorectal Cancer Surgery
**eTable 8.** Multivariate Logistic Regression Analyses for Readmission After Colorectal Cancer Surgery
**eTable 9.** Multivariate Logistic Regression Analyses for Prolonged Stay in Hospital After Colorectal Cancer Surgery
**eFigure 1.** Pairwise AUC Comparisons for All Models
**eFigure 2.** Significant Predictors in Multivariate Logistic Regression Models for Complicated Course
**eFigure 3.** Significant Predictors in Multivariate Logistic Regression Models for ICU Admission
**eFigure 4.** Significant Predictors in Multivariate Logistic Regression Models for Readmission
**eFigure 5.** Significant Predictors in Multivariate Logistic Regression Models for Prolonged Length of Hospital Stay
**eFigure 6.** Most Influential Predictor Variables for Complicated Course
**eFigure 7.** Most Influential Predictor Variables for ICU Admission
**eFigure 8.** Most Influential Predictor Variables for Hospital Readmission
**eFigure 9.** Most Influential Predictor Variables for Prolonged Length of Hospital Stay

This supplementary material has been provided by the authors to give readers additional information about their work.

© 2021 van den Bosch T et al. *JAMA Network Open.*

**eTable 1.** Characteristics of 62 501 Patient Records Included in This Study

| | Total set (n = 62,501) | Missing (n=49,878) | Train set (n = 50,589) | Test set (n = 11,912) | *P* value |
|---|---|---|---|---|---|
| **Sex** | | 21 (0.0%) | | | .003 |
| Male | 35,116 (56.2%) | | 28,278 (55.9%) | 6,838 (57.4%) | |
| Female | 27,364 (43.8%) | | 22,293 (44.1%) | 5,071 (42.6%) | |
| **Age (years)** | | 57 (0.1%) | | | <.001 |
| <60 | 11,071 (17.7%) | | 9,088 (18.0%) | 1,983 (16.6%) | |
| 61-70 | 21,004 (33.6%) | | 16,714 (33.0%) | 4,290 (36.0%) | |
| 71-80 | 20,556 (32.9%) | | 16,603 (32.8%) | 3,953 (33.2%) | |
| ≥80 | 9,813 (15.7%) | | 8,146 (16.1%) | 1,667 (14.0%) | |
| **BMI (kg/m2)** | | 3190 (5.1%) | | | |
| <18.5 | 1,038 (1.7%) | | 856 (1.7%) | 182 (1.5%) | <.001 |
| 18.5-25.0 | 23,907 (38.3%) | | 19,450 (38.4%) | 4,457 (37.4%) | |
| 25.0-30.0 | 23,898 (38.2%) | | 19,350 (38.2%) | 4,548 (38.2%) | |
| ≥30 | 10,468 (16.8%) | | 8,128 (16.1%) | 2,340 (19.6%) | |
| **ASA-score** | | 513 (0.8%) | | | <.001 |
| I | 11,964 (19.1%) | | 9,979 (19.7%) | 1,985 (16.7%) | |
| II | 35,679 (57.1%) | | 28,587 (56.5%) | 7,092 (59.5%) | |
| III | 13,370 (21.4%) | | 10,757 (21.3%) | 2,613 (21.9%) | |
| IV | 949 (1.5%) | | 740 (1.5%) | 209 (1.8%) | |
| V | 26 (0.0%) | | 22 (0.0%) | 4 (0.0%) | |
| **Comorbidities** | | | | | |
| Cardiac | | 259 (0.4%) | | | |
| Angina Pectoris | 2,726 (4.4%) | | 2,218 (4.4%) | 508 (4.3%) | .53 |
| Myocardial infarction | 3,857 (6.2%) | | 3,125 (6.2%) | 732 (6.1%) | .84 |
| Percutaneous transluminal coronary angioplasty (PTCA) | 2,498 (4.0%) | | 1,996 (3.9%) | 502 (4.2%) | .21 |
| Coronary artery bypass grafting (CABG) | 2,274 (3.6%) | | 1,870 (3.7%) | 404 (3.4%) | .10 |
| Valvular heart disease | 2,258 (3.6%) | | 1,802 (3.6%) | 456 (3.8%) | .19 |
| Cardiac valve replacement | 685 (1.1%) | | 408 (0.8%) | 120 (1.0%) | .47 |
| Atrial fibrillation/ atrial flutter | 5,184 (8.3%) | | 4,141 (8.2%) | 1,043 (8.8%) | .06 |
| Cardiac arrhythmia (exclusive atrial fibrillation and flutter) | 2,427 (3.9%) | | 1,952 (3.9%) | 475 (4.0%) | .58 |
| Congestive heart failure | 1,346 (2.1%) | | 1,113 (2.2%) | 233 (2.0%) | .10 |
| Cardiomyopathy | 528 (0.8%) | | 408 (0.8%) | 120 (1.0%) | .04 |
| Medical history of heart transplantation | 9 (0.0%) | | 8 (0.0%) | 1 (0.0%) | .85 |
| Other heart disease | 1,743 (2.8%) | | 1,355 (2.7%) | 388 (3.3%) | .001 |

© 2021 van den Bosch T et al. *JAMA Network Open.*

## eTable 1 continued: Characteristics of 62,501 patient records included in this study

| | Total set | Missing | Train set | Test set | *P* value |
|---|---|---|---|---|---|
| Vascular | | 246 (0.4%) | | | |
| Hypertension | 22,581 (36.1%) | | 18,084 (35.7%) | 4,497 (37.8%) | <.001 |
| Peripheral vascular disease | 1,836 (2.9%) | | 1,464 (2.9%) | 372 (3.1%) | .20 |
| Carotid artery stenosis | 320 (0.5%) | | 255 (0.5%) | 65 (0.5%) | .62 |
| Aortic aneurysm (abdominal/thoracic) | 1,283 (2.1%) | | 1,028 (2.0%) | 255 (2.1%) | .48 |
| Other vascular disease | 1,352 (2.2%) | | 1,097 (2.2%) | 255 (2.1%) | .87 |
| Diabetes mellitus | | 246 (0.4%) | | | |
| Non-insulin-dependent diabetes mellitus | 7,251 (11.6%) | | 5,802 (11.5%) | 1,449 (12.2%) | .05 |
| Insulin-dependent diabetes mellitus | 2,270 (3.6%) | | 1,828 (3.6%) | 442 (3.7%) | .68 |
| Diabetes mellitus with end-organ damage | 273 (0.4%) | | 205 (0.4%) | 68 (0.6%) | .02 |
| Other diabetes mellitus related comorbidity | 175 (0.3%) | | 151 (0.3%) | 24 (0.2%) | .09 |
| Pulmonary | | 82 (0.1%) | | | |
| COPD/asthma | 6,803 (10.9%) | | 5,497 (10.9%) | 1,306 (11.0%) | .79 |
| Pulmonary fibrosis | 83 (0.1%) | | 70 (0.1%) | 13 (0.1%) | .52 |
| History of lung surgery/transplantation | 217 (0.4%) | | 178 (0.4%) | 39 (0.3%) | .75 |
| Other pulmonary disease | 1,578 (2.5%) | | 1,187 (2.3%) | 391 (3.3%) | .01 |
| Neurological | | 71 (0.1%) | | | |
| Transient ischemic attack | 2,661 (4.3%) | | 2,133 (4.2%) | 528 (4.4%) | .30 |
| Cerebrovascular attack | 2,593 (4.2%) | | 2,108 (4.2%) | 485 (4.1%) | .66 |
| Myopathy | 146 (0.2%) | | 118 (0.2%) | 28 (0.2%) | 1.00 |
| Hemiplegia/paraplegia | 119 (0.2%) | | 97 (0.2%) | 22 (0.2%) | .97 |
| Parkinson's disease/ dementia | 857 (1.4%) | | 700 (1.4%) | 157 (1.3%) | .61 |
| Schizophrenia/ major depressive disorder/ psychosis | 947 (1.5%) | | 54 (0.1%) | 17 (0.1%) | .006 |
| Other neurological disease | 2,404 (3.9%) | | 1,934 (3.8%) | 470 (3.9%) | .54 |

© 2021 van den Bosch T et al. *JAMA Network Open.*

**eTable 1 continued: Characteristics of 62,501 patient records included in this study**

| | Total set | Missing | Train set | Test set | *P* value |
|---|---|---|---|---|---|
| Gastrointestinal | | 107 (0.2%) | | | |
| Gastroesophageal reflux disorder / peptic ulcer disease | 2,016 (3.2%) | | 1,629 (3.2%) | 387 (3.2%) | .91 |
| Symptomatic cholelithiasis | 855 (1.4%) | | 628 (1.2%) | 227 (1.9%) | <.001 |
| Pancreatitis (acute/chronic) | 199 (0.3%) | | 158 (0.3%) | 41 (0.3%) | .65 |
| Inflammatory bowel disease (M. Crohn / Ulcerative Colitis) | 556 (0.9%) | | 451 (0.9%) | 105 (0.9%) | .95 |
| Diverticulitis | 789 (1.3%) | | 630 (1.2%) | 159 (1.3%) | .47 |
| Liver disease/ failure (cirrhosis/hepatitis) | 453 (0.7%) | | 376 (0.7%) | 77 (0.6%) | .27 |
| Other gastrointestinal disease | 1,503 (2.4%) | | 1,182 (2.3%) | 321 (2.7%) | .02 |
| Urogenital | | 86 (0.1%) | | | |
| Chronic kidney disease (creatinine <110) | 1,652 (2.6%) | | 1,267 (2.5%) | 385 (3.2%) | <.001 |
| Dialysis-dependent kidney failure | 70 (0.1%) | | 64 (0.1%) | 6 (0.1%) | .04 |
| Medical history of renal surgery/ transplantation | 377 (0.6%) | | 309 (0.6%) | 68 (0.6%) | .66 |
| Pregnant at the time of surgery | 5 (0.0%) | | 5 (0.0%) | 0 (0.0%) | .61 |
| Other urogenital disease | 2,862 (4.6%) | | 2,264 (4.5%) | 598 (5.0%) | .01 |
| Hematologic | | 64 (0.1%) | | | |
| Deep venous thrombosis | 1,111 (1.8%) | | 913 (1.8%) | 198 (1.7%) | .30 |
| Pulmonary embolism | 872 (1.4%) | | 711 (1.4%) | 161 (1.4%) | .68 |
| Blood clotting disorder | 116 (0.2%) | | 91 (0.2%) | 25 (0.2%) | .57 |
| Other hematologic disease | 268 (0.4%) | | 222 (0.4%) | 46 (0.4%) | .47 |
| Immunodeficiency | | 84 (0.1%) | | | |
| Sarcoidosis/ Besnier Boeck | 102 (0.2%) | | 89 (0.2%) | 13 (0.1%) | .13 |
| Rheumatoid Arthritis/ SLE/ scleroderma | 1748 (2.8%) | | 1,405 (2.8%) | 343 (2.9%) | .57 |
| Vasculitis | 27 (0.0%) | | 22 (0.0%) | 5 (0.0%) | 1.00 |
| Other immunodeficiency | 2,654 (4.3%) | | 2,145 (4.2%) | 509 (4.3%) | .91 |

© 2021 van den Bosch T et al. *JAMA Network Open.*

**eTable 1 continued: Characteristics of 62,501 patient records included in this study**

| | Total set | Missing | Train set | Test set | *P* value |
|---|---|---|---|---|---|
| Endocrine | | 72 (0.1%) | | | |
| Hypo-/hyperthyroidism | 2,571 (4.1%) | | 2,056 (4.1%) | 515 (4.3%) | .21 |
| Hypo-/hyperparathyroidism | 205 (0.3%) | | 151 (0.3%) | 54 (0.5%) | .01 |
| Adrenal disease (Addison Disease/ Cushing Syndrome/ Conn syndrome) | 52 (0.0%) | | 36 (0.1%) | 16 (0.1%) | .05 |
| Other endocrine disease | 555 (0.9%) | | 405 (0.8%) | 150 (1.3%) | .01 |
| Infectious | | 22 (0.0%) | | | |
| HIV/AIDS | 29 (0.1%) | | 24 (0.0%) | 5 (0.0%) | .99 |
| Tuberculosis | 172 (0.3%) | | 147 (0.3%) | 25 (0.2%) | .16 |
| Malaria | 30 (0.1%) | | 25 (0.0%) | 5 (0.0%) | .92 |
| Other infectious disease | 274 (0.4%) | | 188 (0.4%) | 86 (0.7%) | <.001 |
| Malignancy | | 350 (0.6%) | | | |
| Current untreated malignancy | 1,605 (2.6%) | | 1,268 (2.5%) | 337 (2.8%) | .05 |
| Curative malignancy treated <5 years ago | 3,086 (4.9%) | | 2,483 (4.9%) | 603 (5.1%) | .59 |
| Curative malignancy treated >5 years ago | 4,076 (6.5%) | | 3,180 (6.3%) | 896 (7.5%) | <.001 |
| Palliative treated and / or distant metastasis | 430 (0.7%) | | 351 (0.7%) | 79 (0.7%) | .73 |
| Other types of cancer | 173 (0.3%) | | 147 (0.3%) | 26 (0.2%) | .20 |
| **Past surgical history** | | 216 (0.4%) | | | |
| History of esophageal, gastric, pancreatic or spleen surgery | 1,018 (1.6%) | | 850 (1.7%) | 168 (1.4%) | .04 |
| History of hepatobiliary surgery | 4,175 (6.7%) | | 3,403 (6.7%) | 772 (6.5%) | .33 |
| History of bowel surgery | 8,402 (13.4%) | | 6,783 (13.4%) | 1,619 (13.6%) | .63 |
| History of bladder-, prostate-, uterine or ovarian surgery | 7,737 (12.4%) | | 6,382 (12.6%) | 1,355 (11.4%) | <.001 |
| Other abdominal surgery in the history | 3,784 (6.1%) | | 3,093 (6.1%) | 691 (5.8%) | .20 |
| **Stoma before surgical resection** | | 216 (0.4%) | | | .71 |
| No | 61,706 (98.7%) | | 49,932 (98.7%) | 11,774 (98.8%) | |
| Ileostomy | 134 (0.2%) | | 111 (0.2%) | 23 (0.2%) | |
| Colostomy | 425 (0.7%) | | 345 (0.7%) | 80 (0.7%) | |
| Type of stoma unknown | 20 (0.0%) | | 18 (0.0%) | 2 (0.0%) | |

© 2021 van den Bosch T et al. *JAMA Network Open.*

## eTable 1 continued: Characteristics of 62,501 patient records included in this study

| | Total set | Missing | Train set | Test set | *P* value |
|---|---|---|---|---|---|
| **Tumor found by screening** | | 0 (0.0%) | | | <.001 |
| No | 56,639 (90.6%) | | 47,008 (92.9%) | 9,631 (80.9%) | |
| Yes | 5,862 (9.4%) | | 3,581 (7.1%) | 2,281 (19.1%) | |
| **Tumor location** | | 65 (0.1%) | | | .001 |
| Colon | 42,910 (68.7%) | | 34,566 (68.3%) | 8,344 (70.0%) | |
| Rectum | 17,477 (28.0%) | | 14,301 (28.3%) | 3,176 (26.7%) | |
| Multiple tumors | 2,049 (3.3%) | | 1,660 (3.3%) | 389 (3.3%) | |
| **Number tumors found by endoscopy** | | 730 (1.2%) | | | .67 |
| 1 | 59,668 (95.5%) | | 48,148 (95.2%) | 11,520 (96.7%) | |
| 2 | 1,930 (3.1%) | | 1,573 (3.1%) | 357 (3.0%) | |
| 3+ | 173 (0.3%) | | 140 (0.3%) | 33 (0.3%) | |
| **T-stage** | | 2,768 (4.4%) | | | <.001 |
| T0 | 155 (0.3%) | | 116 (0.2%) | 39 (0.3%) | |
| T1 | 4,850 (7.8%) | | 3,521 (7.0%) | 1,329 (11.2%) | |
| T2 | 11,471 (18.4%) | | 9,121 (18.0%) | 2,350 (19.7%) | |
| T3 | 34,553 (55.3%) | | 28,376 (56.1%) | 6,177 (51.9%) | |
| T4 | 8,704 (13.9%) | | 7,136 (14.1%) | 1,568 (13.2%) | |
| **M-stage** | | 5,547 (8.9%) | | | <.001 |
| M0 | 50,656 (81.1%) | | 40,663 (80.4%) | 9,993 (83.9%) | |
| M1 | 6,298 (10.1%) | | 5,258 (10.4%) | 1,040 (8.7%) | |
| **Surgical procedure pre resection** | | 47 (0.1%) | | | |
| Stoma | 2,011 (3.2%) | | 1,652 (3.3%) | 359 (3.0%) | .18 |
| Stent | 168 (0.3%) | | 147 (0.3%) | 21 (0.2%) | .04 |
| Metastasectomy | 582 (0.9%) | | 478 (0.9%) | 104 (0.9%) | .50 |
| Appendectomy | 141 (0.2%) | | 111 (0.2%) | 30 (0.3%) | .57 |
| Other | 496 (0.8%) | | 357 (0.7%) | 139 (1.2%) | <.001 |
| **Metastases** | | 0 (0.0%) | | | |
| Liver metastases | 2,015 (3.2%) | | 1,353 (2.7%) | 662 (5.6%) | <.001 |
| Pulmonary metastases | 487 (0.8%) | | 342 (0.7%) | 145 (1.2%) | <.001 |
| Bone metastasis | 34 (0.1%) | | 22 (0.0%) | 12 (0.1%) | .03 |
| Peritoneal metastasis | 432 (0.7%) | | 249 (0.5%) | 183 (1.5%) | <.001 |
| Ovarian metastasis | 69 (0.1%) | | 35 (0.1%) | 34 (0.3%) | <.001 |
| Brain metastases | 10 (0.2%) | | 6 (0.0%) | 4 (0.0%) | .20 |
| Other metastases | 329 (0.5%) | | 226 (0.4%) | 103 (0.9%) | <.001 |

© 2021 van den Bosch T et al. *JAMA Network Open.*

## eTable 1 continued: Characteristics of 62,501 patient records included in this study

| | Total set | Missing | Train set | Test set | *P* value |
|---|---|---|---|---|---|
| **Preoperative MDT** | | 793 (1.3%) | | | <.001 |
| No | 9,040 (14.5%) | | 7,917 (15.6%) | 1,123 (9.4%) | |
| Yes | 52,668 (84.3%) | | 41,891 (82.8%) | 10,777 (90.5%) | |
| **Preoperative tumor complications** | | 0 (0.0%) | | | |
| Fecal peritonitis due to preoperative colorectal perforation | 860 (1.4%) | | 708 (1.4%) | 152 (1.3%) | .32 |
| Preoperative presence of an abscess | 587 (0.9%) | | 469 (0.9%) | 118 (1.0%) | .55 |
| Preoperative bowel obstruction/ ileus due to malignancy | 6,836 (10.9%) | | 5,720 (11.3%) | 1,116 (9.4%) | <.001 |
| Preoperative blood loss/ anemia | 10,755 (17.2%) | | 8,974 (17.7%) | 1,781 (15.0%) | <.001 |
| Other preoperative tumor related complications | 2,710 (4.3%) | | 2,328 (4.6%) | 382 (3.2%) | <.001 |
| **Neoadjuvant radiotherapy** | | 6,630 (10.6%) | | | <.001 |
| None | 43,163 (69.1%) | | 34,726 (68.6%) | 8,437 (70.8%) | |
| Short course | 6,422 (10.3%) | | 5,649 (11.2%) | 773 (6.5%) | |
| Long course without chemotherapy | 459 (0.7%) | | 432 (0.9%) | 27 (0.2%) | |
| Chemoradiation | 5,827 (9.3%) | | 4,822 (9.5%) | 1,005 (8.4%) | |
| **Neoadjuvant chemotherapy** | | 7,708 (12.3%) | | | <.001 |
| No | 51,064 (81.7%) | | 41,459 (82.0%) | 9,605 (80.6%) | |
| Yes | 3,729 (6.0%) | | 3,132 (6.2%) | 597 (5.0%) | |
| **Setting** | | 437 (0.7%) | | | <.001 |
| Elective | 54,051 (86.5%) | | 43,364 (85.7%) | 10,687 (89.7%) | |
| Elective after placing a stent | 347 (0.6%) | | 290 (0.6%) | 57 (0.5%) | |
| Urgent | 3,682 (5.9%) | | 3,110 (6.1%) | 572 (4.8%) | |
| Emergency, direct procedure | 3,984 (6.4%) | | 3,404 (6.7%) | 580 (4.9%) | |
| **Approach** | | 437 (0.7%) | | | <.001 |
| Open | 23,977 (38.4%) | | 21,434 (42.4%) | 2,543 (21.3%) | |
| Laparoscopic | 36,965 (59.1%) | | 28,021 (55.4%) | 8,944 (75.1%) | |
| Transanal endoscopic microsurgery (TEM) | 745 (1.2%) | | 543 (1.1%) | 202 (1.7%) | |
| Transanal local excision (open) | 54 (0.1%) | | 43 (0.1%) | 11 (0.1%) | |
| Transanal minimally invasive surgery | 109 (0.2%) | | 77 (0.2%) | 32 (0.3%) | |

© 2021 van den Bosch T et al. *JAMA Network Open.*

## eTable 1 continued: Characteristics of 62,501 patient records included in this study

| | Total set | Missing | Train set | Test set | *P* value |
|---|---|---|---|---|---|
| Transanal total mesorectal excision (TaTME) | 214 (0.3%) | | 69 (0.1%) | 145 (1.2%) | |
| **Surgical procedure** | | 63 (0.1%) | | | <.001 |
| Ileocecal resection | 471 (0.8%) | | 413 (0.8%) | 58 (0.5%) | |
| (extended) Right hemicolectomy | 19,284 (30.9%) | | 15,428 (30.5%) | 3,856 (32.4%) | |
| Resection of the transverse colon | 1,073 (1.7%) | | 893 (1.8%) | 180 (1.5%) | |
| (extended) Left hemicolectomy | 4,851 (7.8%) | | 3,899 (7.7%) | 952 (8.0%) | |
| (low) Anterior resection/sigmoid resection | 27,805 (44.5%) | | 22,512 (44.5%) | 5,293 (44.4%) | |
| Subtotal colectomy | 1,008 (1.6%) | | 837 (1.7%) | 171 (1.4%) | |
| Abdominoperineal resection | 4,699 (7.5%) | | 3,957 (7.8%) | 742 (6.2%) | |
| Panproctocolectomy | 133 (0.2%) | | 124 (0.2%) | 9 (0.1%) | |
| Local excision | 718 (1.2%) | | 521 (1.0%) | 197 (1.7%) | |
| Local excision follow by an abdominoperineal resection | 64 (0.1%) | | 49 (0.1%) | 15 (0.1%) | |
| Local excision followed by a total mesorectal excision | 164 (0.3%) | | 115 (0.2%) | 49 (0.4%) | |
| Same procedure for multiple tumors | 910 (1.5%) | | 752 (1.5%) | 158 (1.3%) | |
| Two different procedures of which at least 1 procedure for rectal cancer | 207 (0.3%) | | 155 (0.3%) | 52 (0.4%) | |
| Two different colon procedures | 637 (1.0%) | | 514 (1.0%) | 123 (1.0%) | |
| Other surgical procedures | 414 (0.7%) | | 362 (0.7%) | 52 (0.4%) | |
| ***Surgical conversion** | | 0 (0.00%) | | | .02 |
| No | 58,039 (92.9%) | | 47,017 (92.9%) | 11,022 (92.5%) | |
| Early | 3,018 (4.8%) | | 2,387 (4.7%) | 631 (5.3%) | |
| Late | 1,444 (2.3%) | | 1,185 (2.3%) | 259 (2.2%) | |
| ***Reason for conversion** | | 219 (0.4%) | | | .01 |
| Complexity | 952 (1.5%) | | 737 (1.5%) | 215 (1.8%) | |
| Accessibility | 2,997 (4.8%) | | 2,415 (4.8%) | 582 (4.9%) | |
| Preoperative tumor complication | 294 (0.5%) | | 227 (0.4%) | 67 (0.6%) | |

© 2021 van den Bosch T et al. *JAMA Network Open.*

## eTable 1 continued: Characteristics of 62,501 patient records included in this study

| | Total set | Missing | Train set | Test set | *P* value |
|---|---|---|---|---|---|
| ***Additional resection for local ingrowth** | | 2,551 (4.1%) | | | <.001 |
| No | 54,395 (87.0%) | | 43,635 (86.3%) | 10,760 (90.3%) | |
| Limited | 2,428 (3.9%) | | 2,051 (4.1%) | 377 (3.2%) | |
| Extensive | 3,127 (5.0%) | | 2,563 (5.1%) | 564 (4.7%) | |
| *Resection of the omentum | 592 (1.0%) | 0 (0.00%) | 504 (1.0%) | 88 (0.7%) | .01 |
| *Resection of the liver, or RFA | 858 (1.4%) | 0 (0.00%) | 675 (1.3%) | 183 (1.5%) | .10 |
| *Resection of the lung | 30 (0.1%) | 0 (0.00%) | 26 (0.1%) | 4 (0.0%) | .57 |
| *Resection of the peritoneal metastases | 429 (0.7%) | 0 (0.00%) | 358 (0.7%) | 71 (0.6%) | .21 |
| *Lymph node dissection | 284 (0.5%) | 0 (0.00%) | 235 (0.5%) | 49 (0.4%) | .48 |
| *Resection of other abdominal organs | 475 (0.8%) | 0 (0.00%) | 402 (0.8%) | 73 (0.6%) | .05 |
| *Other abdominal resection | 2,345 (3.8%) | 0 (0.00%) | 1,924 (3.8%) | 421 (3.5%) | .17 |
| ***Radio- or chemotherapy during surgery** | | 0 (0.00%) | | | <.001 |
| No | 62,170 (99.5%) | | 50,352 (99.5%) | 11,818 (99.2%) | |
| IORTC | 149 (0.2%) | | 124 (0.2%) | 25 (0.2%) | |
| HIPEC | 182 (0.3%) | | 113 (0.2%) | 69 (0.6%) | |
| ***Intraoperative complications** | | 3,422 (5.5%) | | | <.001 |
| No | 57,730 (92.4%) | | 46,207 (91.3%) | 11,523 (96.7%) | |
| Yes, blood loss for which blood transfusion was required | 360 (0.6%) | | 310 (0.6%) | 50 (0.4%) | |
| Yes, damage of the spleen for which a splenectomy was required | 79 (0.1%) | | 76 (0.2%) | 3 (0.0%) | |
| Yes, damage to the pancreas, liver, d. choledochus, gallbladder | 41 (0.1%) | | 35 (0.1%) | 6 (0.1%) | |
| Yes, damage to the intestine | 509 (0.8%) | | 411 (0.8%) | 98 (0.8%) | |
| Yes, damage to the ureter/urethra | 201 (0.3%) | | 172 (0.3%) | 29 (0.2%) | |
| Yes, damage to the urine bladder | 101 (0.2%) | | 72 (0.1%) | 29 (0.2%) | |
| Yes, damage to the vagina | 39 (0.1%) | | 34 (0.1%) | 5 (0.0%) | |

© 2021 van den Bosch T et al. *JAMA Network Open.*

## eTable 1 continued: Characteristics of 62,501 patient records included in this study

| | Total set | Missing | Train set | Test set | *P* value |
|---|---|---|---|---|---|
| Perforation intra-abdominal during a transanal local excision | 19 (0.0%) | | 17 (0.0%) | 2 (0.0%) | |
| ***Primary anastomosis** | | 1,508 (2.4%) | | | <.001 |
| No | 13,044 (20.9%) | | 10,910 (21.6%) | 2,134 (17.9%) | |
| Yes | 47,949 (76.7%) | | 38,397 (75.9%) | 9,552 (80.2%) | |
| ***Stoma** | | 1,451 (2.3%) | | | <.001 |
| No | 41,485 (66.4%) | | 32,799 (64.8%) | 8,686 (72.9%) | |
| Loop ileostomy | 6,268 (10.0%) | | 5,343 (10.6%) | 925 (7.8%) | |
| End ileostomy | 1,271 (2.0%) | | 1,041 (2.1%) | 230 (1.9%) | |
| Loop colostomy | 1,104 (1.8%) | | 982 (1.9%) | 122 (1.0%) | |
| End colostomy | 10,851 (17.4%) | | 9,137 (18.1%) | 1,714 (14.4%) | |
| Stoma of unknown type | 71 (0.1%) | | 47 (0.1%) | 24 (0.2%) | |
| **Outcomes** | | 0 (0.0%) | | | |
| Mortality | 1693 (2.7%) | | 1,458 (2.9%) | 235 (2.0%) | <.001 |
| Complicated course | 11443 (18.3%) | | 9,579 (18.9%) | 1,864 (15.6%) | <.001 |
| ICU intake | 11931 (19.1%) | | 9,957 (19.7%) | 1,974 (16.6%) | <.001 |
| Hospital readmission | 4496 (7.2%) | | 3,451 (6.8%) | 1,045 (8.8%) | <.001 |
| Prolonged hospital stay | 4874 (7.8%) | | 4,207 (8.3%) | 667 (5.6%) | <.001 |

**eTable 1:** Characteristics of 62,501 patients included in this study**.** Overview of the dataset. Model split of test (column 4) and train (column 5) sets for shown, with the corresponding *P*-value determined by the chi-square test (column 6). Variables indicated with a * are considered intraoperative variables and are excluded from some analyses as indicated in the main text.

© 2021 van den Bosch T et al. *JAMA Network Open.*

**eTable 2.** Machine Learning Model Specifications for Each Outcome

| Outcome | Method | Balancing | Missing flags | Test set AUC (95%CI) |
|---|---|---|---|---|
| **Mortality** | SVM | Yes | Yes | 0.81 (0.79 - 0.84) |
| | SVM | Yes | No | 0.82 (0.79 - 0.85) |
| | RF | Yes* | Yes | 0.82 (0.79 - 0.85) |
| | RF | No | Yes | 0.80 (0.77 - 0.83) |
| | RF | Yes* | No | 0.82 (0.79 - 0.84) |
| | RF | No | No | 0.79 (0.76 - 0.82) |
| | GBM | Yes | Yes | 0.82 (0.79 - 0.85) |
| | GBM | No | Yes | 0.81 (0.78 - 0.84) |
| | GBM | Yes | No | 0.82 (0.79 - 0.84) |
| | GBM | No | No | 0.80 (0.77 - 0.83) |
| | ENR | Yes | Yes | 0.82 (0.79 - 0.85) |
| | ENR | No | Yes | 0.82 (0.79 - 0.85) |
| | ENR | Yes | No | 0.82 (0.79 - 0.85) |
| | ENR | No | No | 0.82 (0.79 - 0.85) |
| | LR | Yes | Yes | 0.80 (0.77 - 0.83) |
| | LR | No* | Yes | 0.81 (0.78 - 0.84) |
| | LR | Yes | No | 0.81 (0.78 - 0.84) |
| | LR | No | No | 0.81 (0.79 - 0.84) |
| **Complicated Course** | RF | Yes* | Yes | 0.67 (0.65 - 0.68) |
| | RF | No | Yes | 0.66 (0.64 - 0.67) |
| | RF | Yes* | No | 0.67 (0.65 - 0.68) |
| | RF | No | No | 0.66 (0.64 - 0.67) |
| | GBM | Yes* | Yes* | 0.68 (0.67 - 0.69) |
| | GBM | No | Yes | 0.66 (0.65 - 0.67) |
| | GBM | Yes | No | 0.67 (0.66 - 0.68) |
| | GBM | No* | No* | 0.68 (0.67 - 0.69) |
| | ENR | Yes | Yes | 0.68 (0.67 - 0.69) |
| | ENR | No* | Yes | 0.68 (0.67 - 0.69) |
| | ENR | Yes | No | 0.68 (0.67 - 0.69) |
| | ENR | No | No | 0.68 (0.67 - 0.69) |
| | LR | Yes | Yes | 0.68 (0.67 - 0.69) |
| | LR | No | Yes | 0.68 (0.67 - 0.69) |
| | LR | Yes | No | 0.68 (0.67 - 0.69) |
| | LR | No | No | 0.68 (0.67 - 0.69) |
| **ICU admission** | RF | Yes* | Yes | 0.72 (0.71 - 0.74) |
| | RF | No | Yes | 0.72 (0.70 - 0.73) |
| | RF | Yes* | No | 0.72 (0.71 - 0.74) |
| | RF | No | No | 0.71 (0.70 - 0.73) |
| | GBM | Yes | Yes* | 0.73 (0.71 - 0.74) |

© 2021 van den Bosch T et al. *JAMA Network Open.*

## eTable 2 continued: Machine learning model specifications for each outcome

| | Method | Balancing | Missing flags | Test set AUC (95%CI) |
|---|---|---|---|---|
| | GBM | No* | Yes* | 0.74 (0.73 - 0.75) |
| | GBM | Yes | No | 0.72 (0.70 - 0.73) |
| | GBM | No* | No | 0.74 (0.72 - 0.75) |
| | ENR | Yes | Yes* | 0.74 (0.72 - 0.75) |
| | ENR | No | Yes | 0.74 (0.72 - 0.75) |
| | ENR | Yes | No | 0.73 (0.72 - 0.75) |
| | ENR | No | No | 0.73 (0.72 - 0.75) |
| | LR | Yes | Yes | 0.74 (0.72 - 0.75) |
| | LR | No | Yes | 0.74 (0.72 - 0.75) |
| | LR | Yes | No | 0.73 (0.72 - 0.75) |
| | LR | No | No | 0.73 (0.72 - 0.75) |
| **Prolonged stay** | RF | Yes* | Yes* | 0.71 (0.69 - 0.73) |
| | RF | No | Yes | 0.69 (0.67 - 0.71) |
| | RF | Yes | No | 0.70 (0.68 - 0.72) |
| | RF | No | No | 0.70 (0.68 - 0.72) |
| | GBM | Yes | Yes | 0.71 (0.69 - 0.73) |
| | GBM | No | Yes | 0.70 (0.68 - 0.72) |
| | GBM | Yes* | No | 0.71 (0.69 - 0.73) |
| | GBM | No | No | 0.71 (0.69 - 0.73) |
| | ENR | Yes | Yes | 0.71 (0.69 - 0.73) |
| | ENR | No | Yes | 0.71 (0.69 - 0.72) |
| | ENR | Yes | No | 0.71 (0.69 - 0.73) |
| | ENR | No | No | 0.71 (0.69 - 0.72) |
| | LR | Yes | Yes | 0.70 (0.68 - 0.72) |
| | LR | No | Yes | 0.70 (0.68 - 0.72) |
| | LR | Yes | No | 0.70 (0.68 - 0.72) |
| | LR | No | No | 0.70 (0.68 - 0.72) |
| **Readmission** | RF | Yes* | Yes | 0.63 (0.61 - 0.65) |
| | RF | No | Yes | 0.61 (0.59 - 0.62) |
| | RF | Yes* | No | 0.63 (0.61 - 0.65) |
| | RF | No | No | 0.60 (0.59 - 0.62) |
| | GBM | Yes* | Yes | 0.63 (0.62 - 0.65) |
| | GBM | No | Yes | 0.61 (0.59 - 0.63) |
| | GBM | Yes | No | 0.62 (0.61 - 0.64) |
| | GBM | No | No* | 0.62 (0.61 - 0.64) |
| | ENR | Yes | Yes* | 0.62 (0.60 - 0.64) |
| | ENR | No | Yes | 0.62 (0.61 - 0.64) |
| | ENR | Yes | No | 0.61 (0.59 - 0.63) |
| | ENR | No* | No | 0.62 (0.61 - 0.64) |

© 2021 van den Bosch T et al. *JAMA Network Open.*

## eTable 2 continued: Machine learning model specifications for each outcome

| | Method | Balancing | Missing flags | Test set AUC (95%CI) |
|---|---|---|---|---|
| | LR | Yes | Yes | 0.62 (0.60 - 0.64) |
| | LR | No | Yes | 0.62 (0.60 - 0.64) |
| | LR | Yes | No | 0.62 (0.60 - 0.63) |
| | LR | No | No | 0.62 (0.60 - 0.64) |

**eTable 2:** AUC scores for all machine learning models. AUC scores for Elastic Net Regression, Random Forest, Gradient Boosting Models and Support Vector Machine. The best performing model for each response is highlighted in bold. Significantly different AUC (deLong *P*<.05) is denoted by a * when a model with/without balancing or with/without missing flags performs better than the model without/with that method for data handling.

© 2021 van den Bosch T et al. *JAMA Network Open.*

## **eTable 3.** Multicollinearity in the DCRA Dataset

| **Variable** | **VIF** |
|---|---|
| Procedure: 2 equal procedures for multiple tumors | 5.21 |
| Approach: Transanal endoscopic microsurgery (TEM) | 5.43 |
| Procedure: Local excision | 5.61 |
| Tumor location: rectum | 6.02 |
| Number of tumors found: 2 | 35.43 |
| Tumor location: multiple tumors | 45.77 |

**eTable 3 | Multicollinearity in the DCRA dataset** All variables with Variance Inflation Factor >5 in the analyzed dataset. In total, 103 variables were analyzed. Categorical variables are transformed into a single variable for each level, totaling to 144 features of which 7 have a VIF>5. All variables with VIF>5 are levels of a categorical, indicating a low overall multicollinearity.

© 2021 van den Bosch T et al. *JAMA Network Open.*

**eTable 4.** Goodness-of-Fit of Multivariate Logistic Regression Model

| **Model** | **AIC** | **Log-likelihood** | ***P* value** |
|---|---|---|---|
| Full model | 12906 | -6309 | |
| Ground model | 15561 | -7779 | $< 10^{-300}$ |
| Case-mix model (no CCI) | 13271 | -6615 | $8.5 \cdot 10^{-66}$ |
| Case-mix model (including CCI variables) | 13144 | -6524 | $7.11 \cdot 10^{-44}$ |

**eTable 4:** Goodness of fit of multivariable logistic regression model. Comparison of multivariable logistic regression models on mortality containing all variables (full model), no variables (ground model), the DCRA case-mix variables without CCI and the DCRA case-mix variables combined with all variables in the CCI. *P*-values were calculated by the likelihood-ratio test of each model relative to the model containing all variables. The Akaike Information Criterium (AIC) penalizes model goodness of fit with the number of variables in a model. Low AIC indicates good model performance.

© 2021 van den Bosch T et al. *JAMA Network Open.*

**eTable 5.** Multivariate Logistic Regression Analyses for Mortality After Colorectal Cancer Surgery

| **Characteristics** | | **β** | **95% CI** | ***P* value** |
|---|---|---|---|---|
| **(Intercept)** | | -5.24 | -5.63 - -4.85 | <.001 |
| **Sex** | Male | Ref. | | |
| | Female | -0.18 | -0.29 - -0.06 | .003 |
| **Comorbidities** | Angina Pectoris | 0.23 | 0.04 - 0.42 | .02 |
| | Myocardial infarction | 0.22 | 0.05 - 0.39 | .01 |
| | Percutaneous transluminal coronary angioplasty (PTCA) | -0.14 | -0.37 - 0.09 | .22 |
| | Coronary artery bypass grafting (CABG) | 0.07 | -0.15 - 0.28 | .54 |
| | Valvular heart disease | -0.15 | -0.37 - 0.06 | .16 |
| | Cardiac valve replacement | 0.18 | -0.17 - 0.52 | .31 |
| | Atrial fibrillation/ atrial flutter | 0.21 | 0.07 - 0.36 | .003 |
| | Cardiac arrhythmia (exclusive atrial fibrillation and flutter) | 0.16 | -0.04 - 0.37 | .12 |
| | Congestive heart failure | 0.22 | 0.01 - 0.44 | .04 |
| | Cardiomyopathy | 0.29 | -0.07 - 0.65 | .12 |
| | Medical history of heart transplantation | 1.19 | -0.96 - 3.20 | .29 |
| | Other heart disease | 0.16 | -0.08 - 0.40 | .18 |
| | Hypertension | 0.02 | -0.09 - 0.13 | .77 |
| | Peripheral vascular disease | 0.40 | 0.19 - 0.60 | <.001 |
| | Carotid artery stenosis | 0.20 | -0.26 - 0.66 | .40 |
| | Aortic aneurysm (abdominal/thoracic) | 0.08 | -0.18 - 0.35 | .54 |
| | Other vascular disease | -0.06 | -0.38 - 0.25 | .70 |
| | Non-insulin-dependent diabetes mellitus | 0.12 | -0.03 - 0.26 | .11 |
| | Insulin-dependent diabetes mellitus | 0.27 | 0.04 - 0.49 | .02 |
| | Diabetes mellitus with end-organ damage | -0.15 | -0.70 - 0.40 | .59 |
| | Other diabetes mellitus related comorbidity | 0.59 | -0.06 - 1.23 | .07 |
| | COPD/asthma | 0.40 | 0.27 - 0.53 | <.001 |
| | Pulmonary fibrosis | 0.23 | -0.73 - 1.19 | .63 |
| | History of lung surgery/transplantation | 0.88 | 0.38 - 1.39 | <.001 |
| | Other pulmonary disease | 0.24 | -0.01 - 0.50 | .06 |
| | Transient ischemic attack | 0.20 | 0.00 - 0.40 | .04 |
| | Cerebrovascular attack | 0.25 | 0.07 - 0.44 | .008 |
| | Myopathy | 0.10 | -0.81 - 1.01 | .83 |
| | Hemiplegia/ paraplegia | 0.02 | -0.93 - 0.97 | .97 |
| | Parkinson’s disease/ dementia | 0.56 | 0.30 - 0.82 | <.001 |
| | Schizophrenia/ major depressive disorder/ psychosis | 0.46 | 0.12 - 0.79 | .007 |
| | Other neurological disease | 0.14 | -0.09 - 0.36 | .23 |
| | Gastroesophageal reflux disorder / peptic ulcer disease | -0.25 | -0.53 - 0.03 | .07 |
| | Symptomatic cholelithiasis | -0.07 | -0.48 - 0.33 | .72 |
| | Pancreatitis (acute/chronic) | -0.97 | -2.15 - 0.22 | .11 |
| | Inflammatory bowel disease (M. Crohn/ Ulcerative Colitis) | -0.12 | -0.72 - 0.48 | .69 |
| | Diverticulitis | -0.01 | -0.41 - 0.38 | .94 |
| | Liver disease/ failure (cirrhosis/hepatitis) | 0.94 | 0.55 - 1.34 | <.001 |
| | Other gastrointestinal disease | 0.11 | -0.19 - 0.40 | .47 |
| | Chronic kidney disease (creatinine <110) | 0.37 | 0.16 - 0.57 | <.001 |
| | Dialysis-dependent kidney failure | 0.67 | -0.10 - 1.44 | .08 |
| | Medical history of renal surgery/ transplantation | 0.32 | -0.20 - 0.83 | .22 |
| | Pregnant at the time of surgery | -13.78 | -3296.82 - 3269.26 | .99 |
| | Other urogenital disease | -0.09 | -0.31 - 0.13 | .41 |
| | Deep venous thrombosis | 0.09 | -0.25 - 0.42 | .61 |
| | Pulmonary embolism | 0.14 | -0.21 - 0.50 | .43 |
| | Blood clotting disorder | -0.19 | -1.24 - 0.86 | .72 |
| | Other hematologic disease | 0.18 | -0.49 - 0.86 | .59 |

© 2021 van den Bosch T et al. *JAMA Network Open.*

**eTable 5 continued: Multivariate logistic regression analyses for mortality after colorectal cancer surgery**

| Characteristics | | β | 95% CI | *P* value |
|---|---|---|---|---|
| | Sarcoidosis/ Besnier Boeck | -0.74 | -2.74 - 1.27 | .47 |
| | Rheumatoid Arthritis/ SLE/ scleroderma | -0.04 | -0.31 - 0.24 | .80 |
| | Vasculitis | 0.50 | -1.01 - 2.02 | .51 |
| | Other immunodeficiency | 0.05 | -0.17 - 0.28 | .65 |
| | Hypo-/hyperthyroidism | -0.06 | -0.31 - 0.18 | .60 |
| | Hypo-/hyperparathyroidism | -0.05 | -0.77 - 0.68 | .90 |
| Adrenal disease (Addison Disease/ Cushing Syndrome/ Conn syndrome) | | -13.69 | -1028.69 - 1001.31 | .98 |
| | Other endocrine disease | -0.49 | -1.06 - 0.08 | .09 |
| | HIV/AIDS | 0.52 | -1.54 - 2.58 | .62 |
| | Tuberculosis | 0.01 | -0.80 - 0.82 | .98 |
| | Malaria | -14.66 | -1345.42 - 1316.10 | .98 |
| | Other infectious disease | 0.30 | -0.28 - 0.88 | .31 |
| | Current untreated malignancy | 0.45 | 0.20 - 0.70 | <.001 |
| | Curative malignancy treated <5 years ago | -0.03 | -0.26 - 0.19 | .76 |
| | Curative malignancy treated >5 years ago | 0.15 | -0.03 - 0.32 | .10 |
| | Palliative treated and / or distant metastasis | 0.26 | -0.18 - 0.70 | .25 |
| | Other types of cancer | 0.80 | 0.25 - 1.34 | .004 |
| **Surgical history** | History of esophageal, gastric, pancreatic or spleen surgery | 0.06 | -0.29 - 0.41 | .73 |
| | History of hepatobiliary surgery | 0.13 | -0.06 - 0.32 | .19 |
| | History of bowel surgery | -0.01 | -0.16 - 0.14 | .88 |
| | History of bladder-, prostate-, uterine or ovarian surgery | -0.18 | -0.35 - -0.01 | .04 |
| | Other abdominal surgery in the history | 0.02 | -0.18 - 0.22 | .84 |
| **Stoma before surgical resection** | No stoma | Ref. | | |
| | Ileostomy | -13.8 | -643.53 - 616.01 | .97 |
| | Colostomy | -0.25 | -0.87 - 0.38 | .44 |
| | Type unknown | 0.10 | -2.00 - 2.21 | .92 |
| **Number of tumors found by endoscopy** | 1 | Ref. | | |
| | 2 | -0.1 | -1.60 - 1.50 | .95 |
| | 3+ | -0.22 | -1.83 - 1.39 | .79 |
| **Preoperative MDT** | | -0.14 | -0.30 - 0.01 | .07 |
| **Preoperative surgical procedure** | Stoma | 0.03 | -0.32 - 0.39 | .87 |
| | Stent | 0.20 | -0.71 - 1.12 | .66 |
| | Metastasectomy | -1.32 | -2.50 - -0.14 | .03 |
| | Appendectomy | -0.01 | -1.44 - 1.42 | .99 |
| | Other | -0.04 | -0.67 - 0.60 | .91 |
| **Neoadjuvant radiotherapy** | No neoadjuvant radiotherapy | Ref. | | |
| | Short course | -0.03 | -0.32 - 0.25 | .81 |
| | Long course without chemotherapy | 0.32 | -0.09 - 0.73 | .12 |
| | Chemoradiation | -0.35 | -0.74 - 0.04 | .08 |
| **Neoadjuvant chemotherapy** | | 0.05 | -0.25 - 0.35 | .73 |
| **ASA score** | I | Ref. | | |
| | II | 0.69 | 0.42 - 0.97 | <.001 |
| | III | 1.36 | 1.07 - 1.64 | <.001 |
| | IV | 2.20 | 1.86 - 2.53 | <.001 |
| | V | 3.18 | 2.24 - 4.12 | <.001 |
| **Setting** | Elective | Ref. | | |
| | Elective after placing a stent | -0.25 | -1.17 - 0.67 | .59 |
| | Urgent | 0.42 | 0.20 - 0.63 | <.001 |
| | Emergency, direct procedure | 0.61 | 0.37 - 0.85 | <.001 |
| **Tumor complications** | No tumor complications | Ref. | | |
| | Fecal peritonitis due to preoperative colorectal perforation | 0.92 | 0.64 - 1.20 | <.001 |

© 2021 van den Bosch T et al. *JAMA Network Open.*

## eTable 5 continued: Multivariate logistic regression analyses for mortality after colorectal cancer surgery

| Characteristics | | β | 95% CI | *P* value |
|---|---|---|---|---|
| | Preoperative presence of an abscess | 0.04 | -0.41 - 0.49 | .86 |
| | Preoperative bowel obstruction/ ileus due to malignancy | 0.00 | -0.19 - 0.20 | .97 |
| | Preoperative blood loss/ anemia | 0.05 | -0.09 - 0.19 | .47 |
| | Other preoperative tumor related complications | 0.07 | -0.17 - 0.31 | .55 |
| **Approach** | Open | Ref. | | |
| | Laparoscopic | -0.52 | -0.64 - -0.39 | <.001 |
| | Transanal endoscopic microsurgery (TEM) | -0.21 | -1.63 - 1.23 | .78 |
| | Transanal local excision (open) | -1.07 | -3.17 - 1.03 | .31 |
| | Transanal minimally invasive surgery (TAMIS) | -13.09 | -707.27 - 681.09 | .97 |
| | Transanal total mesorectal excision (TaTME) | -13.49 | -512.88 - 485.91 | .96 |
| **Metastasis** | Liver metastasis | -0.10 | -0.40 - 0.21 | .53 |
| | Pulmonary metastasis | -0.39 | -0.95 - 0.17 | .16 |
| | Bone metastasis | 1.69 | 0.67 - 2.7 | .001 |
| | Peritoneal metastasis | 0.17 | -0.34 - 0.67 | .51 |
| | Ovarian metastasis | 0.31 | -1.25 - 1.86 | .70 |
| | Brain metastasis | -13.78 | -2290.19 - 2262.63 | .99 |
| | Other metastasis | -0.33 | -1.00 - 0.33 | .32 |
| **Age** | 60-70 | Ref. | | |
| | <60 | -0.36 | -0.62 - -0.10 | .006 |
| | ≥80 | 1.24 | 1.08 - 1.40 | <.001 |
| | 70-80 | 0.59 | 0.44 - 0.74 | <.001 |
| **BMI** | 18.5-25.0 | Ref. | | |
| | <18.5 | 0.45 | 0.17 - 0.72 | .001 |
| | ≥30.0 | -0.04 | -0.19 - 0.11 | .63 |
| | 25.0-30.0 | -0.12 | -0.24 - -0.00 | .04 |
| **Tumor location** | Colon | Ref. | | |
| | Rectum | 0.25 | -0.04 - 0.54 | .09 |
| | Multiple tumors | 0.09 | -1.56 - 1.74 | .91 |
| **Tumor found by screening** | | -0.17 | -0.47 - 0.12 | .025 |
| **Procedure** | (low) Anterior/ sigmoid resection | Ref. | | |
| | Ileocecal resection | 0.40 | -0.01 - 0.81 | .06 |
| | (extended) Right hemicolectomy | 0.20 | 0.06 - 0.34 | .005 |
| | Local excision followed by a total mesorectal excision | 0.32 | -1.76 - 2.40 | .76 |
| | Same procedure for multiple tumors | 0.18 | -0.58 - 0.93 | .64 |
| | Two different procedures of which at least 1 procedure for rectal cancer | 1.23 | 0.34 - 2.12 | .006 |
| | Two different colon procedures | 0.73 | -0.01 - 1.47 | .05 |
| | Transversectomy | 0.44 | 0.12 - 0.75 | .006 |
| | (extended) Left hemicolectomy | 0.29 | 0.09 - 0.49 | .004 |
| | Subtotal colectomy | 0.87 | 0.54 - 1.20 | <.001 |
| | Abdominoperineal resection | 0.16 | -0.11 - 0.43 | .23 |
| | Panproctocolectomy | 1.28 | 0.53 - 2.02 | <.001 |
| | Other | 0.39 | -0.13 - 0.91 | .14 |
| | Local excision | -1.48 | -3.20 - 0.23 | .09 |
| | Local excision followed by an abdominoperineal resection | -0.57 | -2.08 - 0.94 | .45 |
| **M-stage** | M0 | Ref. | | |
| | M1 | 0.52 | 0.34 - 0.69 | <.001 |
| **T-stage** | T1 | Ref. | | |
| | T2 | -0.11 | -0.35 - 0.13 | .36 |
| | T3 | -0.09 | -0.31 - 0.14 | .45 |
| | T4 | 0.05 | -0.20 - 0.30 | .69 |
| | T0 | -0.06 | -1.28 - 1.16 | .92 |

**eTable 5:** Logistic regression coefficients for 30-day mortality on 62,501 patient records. The *P*-values are given as calculated by the Wald test. When no reference level is reported, variables are binary and the reference level is absence of that variable.

© 2021 van den Bosch T et al. *JAMA Network Open.*

**eTable 6.** Multivariate Logistic Regression Analyses for Complicated Course After Colorectal Cancer Surgery

| Characteristics | | β | 95% CI | *P* value |
|---|---|---|---|---|
| **(Intercept)** | | -2.11 | -2.24 - -1.97 | <.001 |
| **Sex** | Male | Ref. | | |
| | Female | -0.42 | -0.47 - -0.37 | <.001 |
| **Comorbidities** | Angina Pectoris | 0.03 | -0.07 - 0.13 | .60 |
| | Myocardial infarction | 0.05 | -0.04 - 0.14 | .23 |
| | Percutaneous transluminal coronary angioplasty (PTCA) | -0.06 | -0.17 - 0.05 | .30 |
| | Coronary artery bypass grafting (CABG) | -0.04 | -0.15 - 0.07 | .47 |
| | Valvular heart disease | -0.06 | -0.17 - 0.05 | .28 |
| | Cardiac valve replacement | 0.36 | 0.18 - 0.54 | <.001 |
| | Atrial fibrillation/ atrial flutter | 0.12 | 0.05 - 0.19 | .002 |
| | Cardiac arrhythmia (exclusive atrial fibrillation and flutter) | 0.08 | -0.03 - 0.18 | .16 |
| | Congestive heart failure | 0.22 | 0.09 - 0.35 | <.001 |
| | Cardiomyopathy | 0.20 | -0.00 - 0.40 | .05 |
| | Medical history of heart transplantation | 0.79 | -0.66 - 2.24 | .28 |
| | Other heart disease | 0.02 | -0.10 - 0.14 | .75 |
| | Hypertension | -0.01 | -0.05 - 0.04 | .79 |
| | Peripheral vascular disease | 0.19 | 0.08 - 0.31 | <.001 |
| | Carotid artery stenosis | 0.03 | -0.24 - 0.30 | .81 |
| | Aortic aneurysm (abdominal/thoracic) | 0.16 | 0.02 - 0.29 | .02 |
| | Other vascular disease | 0.03 | -0.11 - 0.17 | .66 |
| | Non-insulin-dependent diabetes mellitus | -0.03 | -0.09 - 0.04 | .42 |
| | Insulin-dependent diabetes mellitus | -0.02 | -0.13 - 0.09 | .71 |
| | Diabetes mellitus with end-organ damage | -0.19 | -0.50 - 0.12 | .23 |
| | Other diabetes mellitus related comorbidity | 0.12 | -0.25 - 0.49 | .52 |
| | COPD/asthma | 0.27 | 0.20 - 0.33 | <.001 |
| | Pulmonary fibrosis | 0.61 | 0.13 - 1.09 | .01 |
| | History of lung surgery/transplantation | 0.08 | -0.25 - 0.40 | .63 |
| | Other pulmonary disease | 0.04 | -0.09 - 0.17 | .52 |
| | Transient ischemic attack | 0.11 | 0.02 - 0.21 | .02 |
| | Cerebrovascular attack | 0.03 | -0.07 - 0.14 | .50 |
| | Myopathy | 0.34 | -0.05 - 0.74 | .09 |
| | Hemiplegia/ paraplegia | 0.05 | -0.39 - 0.49 | .83 |
| | Parkinson’s disease/ dementia | 0.24 | 0.08 - 0.40 | .003 |
| | Schizophrenia/ major depressive disorder/ psychosis | 0.07 | -0.10 - 0.24 | .44 |
| | Other neurological disease | 0.13 | 0.02 - 0.23 | .01 |
| | Gastroesophageal reflux disorder / peptic ulcer disease | 0.06 | -0.06 - 0.18 | .31 |
| | Symptomatic cholelithiasis | 0.17 | -0.01 - 0.35 | .05 |
| | Pancreatitis (acute/chronic) | 0.26 | -0.08 - 0.60 | .13 |
| | Inflammatory bowel disease (M. Crohn/ Ulcerative Colitis) | -0.09 | -0.31 - 0.14 | .45 |
| | Diverticulitis | 0.12 | -0.06 - 0.29 | .20 |
| | Liver disease/ failure (cirrhosis/hepatitis) | 0.38 | 0.16 - 0.60 | <.001 |
| | Other gastrointestinal disease | 0.03 | -0.10 - 0.17 | .64 |
| | Chronic kidney disease (creatinine <110) | 0.16 | 0.04 - 0.28 | .01 |
| | Dialysis-dependent kidney failure | 0.48 | -0.05 - 1.02 | .07 |
| | Medical history of renal surgery/ transplantation | 0.10 | -0.16 - 0.37 | .43 |
| | Pregnant at the time of surgery | 0.35 | -1.90 - 2.60 | .76 |
| | Other urogenital disease | 0.02 | -0.08 - 0.11 | .75 |
| | Deep venous thrombosis | -0.03 | -0.19 - 0.13 | .74 |
| | Pulmonary embolism | 0.20 | 0.03 - 0.37 | .02 |
| | Blood clotting disorder | -0.18 | -0.67 - 0.32 | .48 |

© 2021 van den Bosch T et al. *JAMA Network Open.*

## eTable 6 continued: Multivariate logistic regression analyses for complicated course after colorectal cancer surgery

| Characteristics | | β | 95% CI | *P* value |
|---|---|---|---|---|
| | Other hematologic disease | -0.05 | -0.37 - 0.27 | .75 |
| | Sarcoidosis/ Besnier Boeck | 0.35 | -0.13 - 0.83 | .14 |
| | Rheumatoid Arthritis/ SLE/ scleroderma | 0.10 | -0.02 - 0.23 | .11 |
| | Vasculitis | 0.22 | -0.66 - 1.09 | .62 |
| | Other immunodeficiency | 0.13 | 0.02 - 0.23 | .01 |
| | Hypo-/hyperthyroidism | 0.03 | -0.08 - 0.14 | .56 |
| | Hypo-/hyperparathyroidism | -0.09 | -0.46 - 0.28 | .61 |
| | Adrenal disease (Addison Disease/ Cushing Syndrome/ Conn syndrome) | 0.60 | -0.04 - 1.24 | .06 |
| | Other endocrine disease | 0.06 | -0.17 - 0.28 | .63 |
| | HIV/AIDS | 0.41 | -0.42 - 1.25 | .33 |
| | Tuberculosis | -0.03 | -0.42 - 0.37 | .90 |
| | Malaria | -0.45 | -1.45 - 0.55 | .37 |
| | Other infectious disease | -0.04 | -0.35 - 0.28 | .82 |
| | Current untreated malignancy | 0.22 | 0.09 - 0.34 | <.001 |
| | Curative malignancy treated <5 years ago | 0.04 | -0.06 - 0.14 | .42 |
| | Curative malignancy treated >5 years ago | 0.11 | 0.03 - 0.20 | .008 |
| | Palliative treated and / or distant metastasis | 0.02 | -0.22 - 0.25 | .90 |
| | Other types of cancer | 0.02 | -0.35 - 0.38 | .94 |
| **Surgical history** | History of esophageal, gastric, pancreatic or spleen surgery | 0.14 | -0.01 - 0.30 | .07 |
| | History of hepatobiliary surgery | 0.10 | 0.01 - 0.18 | .03 |
| | History of bowel surgery | 0.11 | 0.05 - 0.18 | <.001 |
| | History of bladder-, prostate-, uterine or ovarian surgery | 0.01 | -0.06 - 0.08 | .72 |
| | Other abdominal surgery in the history | -0.04 | -0.13 - 0.05 | .43 |
| **Stoma before surgical resection** | No stoma | Ref. | | |
| | Ileostomy | 0.38 | -0.01 - 0.78 | .05 |
| | Colostomy | -0.13 | -0.38 - 0.12 | .31 |
| | Type unknown | 0.55 | -0.42 - 1.52 | .26 |
| **Number of tumors found by endoscopy** | 1 | Ref. | | |
| | 2 | -0.27 | -0.99 - 0.44 | .45 |
| | 3+ | -0.22 | -0.97 - 0.54 | .57 |
| **Preoperative MDT** | | -0.08 | -0.15 - -0.00 | .04 |
| **Preoperative surgical procedure** | Stoma | -0.13 | -0.26 - -0.00 | .04 |
| | Stent | 0.16 | -0.27 - 0.59 | .45 |
| | Metastasectomy | -0.38 | -0.62 - -0.13 | .002 |
| | Appendectomy | -0.19 | -0.68 - 0.30 | .44 |
| | Other | 0.28 | 0.06 - 0.50 | .01 |
| **Neoadjuvant radiotherapy** | No neoadjuvant radiotherapy | Ref. | | |
| | Short course | 0.27 | 0.16 - 0.38 | <.001 |
| | Long course without chemotherapy | 0.16 | -0.01 - 0.32 | .06 |
| | Chemoradiation | 0.09 | -0.04 - 0.22 | .17 |
| **Neoadjuvant chemotherapy** | | 0.15 | 0.06 - 0.25 | .002 |
| **ASA score** | I | Ref. | | |
| | II | 0.14 | 0.07 - 0.20 | <.001 |
| | III | 0.50 | 0.42 - 0.59 | <.001 |
| | IV | 0.98 | 0.82 - 1.14 | <.001 |
| | V | 1.14 | 0.29 - 1.98 | .008 |
| **Setting** | Elective | Ref. | | |
| | Elective after placing a stent | -0.10 | -0.42 - 0.22 | .54 |
| | Urgent | 0.24 | 0.14 - 0.35 | <.001 |
| | Emergency, direct procedure | 0.38 | 0.26 - 0.50 | <.001 |
| **Tumor complications** | Fecal peritonitis due to preoperative colorectal perforation | 0.70 | 0.53 - 0.87 | <.001 |
| | Preoperative presence of an abscess | 0.39 | 0.20 - 0.59 | <.001 |

© 2021 van den Bosch T et al. *JAMA Network Open.*

## eTable 6 continued: Multivariate logistic regression analyses for complicated course after colorectal cancer surgery

| Characteristics | | β | 95% CI | *P* value |
|---|---|---|---|---|
| | Preoperative bowel obstruction/ ileus due to malignancy | 0.10 | 0.00 - 0.19 | .05 |
| | Preoperative blood loss/ anemia | 0.10 | 0.04 - 0.16 | <.001 |
| | Other preoperative tumor related complications | 0.19 | 0.09 - 0.29 | <.001 |
| **Approach** | Open | Ref. | | |
| | Laparoscopic | -0.37 | -0.42 - -0.32 | <.001 |
| | Transanal endoscopic microsurgery (TEM) | -0.75 | -1.30 - -0.20 | .007 |
| | Transanal local excision (open) | -0.24 | -0.96 - 0.48 | .51 |
| | Transanal minimally invasive surgery (TAMIS) | -0.71 | -1.47 - 0.04 | .06 |
| | Transanal total mesorectal excision (TaTME) | -0.11 | -0.44 - 0.23 | .54 |
| **Metastasis** | Liver metastasis | -0.04 | -0.18 - 0.10 | .59 |
| | Pulmonary metastasis | -0.28 | -0.52 - -0.03 | .03 |
| | Bone metastasis | 0.55 | -0.19 - 1.30 | .14 |
| | Peritoneal metastasis | 0.19 | -0.06 - 0.43 | .13 |
| | Ovarian metastasis | 0.54 | -0.04 - 1.122 | .06 |
| | Brain metastasis | -0.04 | -1.65 - 1.57 | .96 |
| | Other metastasis | 0.17 | -0.10 - 0.44 | .20 |
| **Age** | 60-70 | Ref. | | |
| | <60 | -0.05 | -0.11 - 0.02 | .19 |
| | ≥80 | 0.32 | 0.25 - 0.39 | <.001 |
| | 70-80 | 0.12 | 0.06 - 0.17 | <.001 |
| **BMI** | 18.5-25.0 | Ref. | | |
| | <18.5 | 0.13 | -0.01 - 0.27 | .07 |
| | ≥30.0 | 0.17 | 0.11 - 0.23 | <.001 |
| | 25.0-30.0 | 0.03 | -0.02 - 0.08 | .28 |
| **Tumor location** | Colon | Ref. | | |
| | Rectum | 0.50 | 0.38 - 0.61 | <.001 |
| | Multiple tumors | 0.28 | -0.48 - 1.05 | .46 |
| **Tumor found by screening** | | -0.15 | -0.24 - -0.06 | <.001 |
| **Procedure** | (low) Anterior/sigmoid resection | Ref. | | |
| | Ileocecal resection | -0.05 | -0.28 - 0.19 | .70 |
| | (extended) Right hemicolectomy | 0.09 | 0.03 - 0.15 | .005 |
| | Local excision followed by a total mesorectal excision | 0.26 | -0.35 - 0.87 | .39 |
| | Same procedure for multiple tumors | 0.35 | 0.01 - 0.70 | .04 |
| | Two different procedures of which at least 1 procedure for rectal cancer | 0.72 | 0.29 - 1.16 | .001 |
| | Two different colon procedures | 0.64 | 0.29 - 0.99 | <.001 |
| | Transversectomy | 0.15 | -0.02 - 0.31 | .07 |
| | (extended) Left hemicolectomy | 0.31 | 0.22 - 0.39 | <.001 |
| | Subtotal colectomy | 1.10 | 0.93 - 1.26 | <.001 |
| | Abdominoperineal resection | -0.12 | -0.21 - -0.04 | .005 |
| | Panproctocolectomy | 0.74 | 0.35 - 1.13 | <.001 |
| | Other | 0.00 | -0.26 - 0.25 | .97 |
| | Local excision | -1.46 | -2.09 - -0.83 | <.001 |
| | Local excision followed by an abdominoperineal resection | -0.06 | -0.50 - 0.38 | .79 |
| **M-stage** | M0 | Ref. | | |
| | M1 | 0.08 | -0.01 - 0.17 | .06 |
| **T-stage** | T1 | Ref. | | |
| | T2 | 0.10 | 0.00 - 0.19 | .05 |
| | T3 | 0.16 | 0.07 - 0.250 | <.001 |
| | T4 | 0.35 | 0.25 - 0.46 | <.001 |
| | T0 | 0.36 | -0.09 - 0.81 | .11 |

**eTable 6:** Logistic regression coefficients for complicated course on 62,501 patient records. The *P*-values are given as calculated by the Wald test. When no reference level is reported, variables are binary and the reference level is absence of that variable.

© 2021 van den Bosch T et al. *JAMA Network Open.*

**eTable 7.** Multivariate Logistic Regression Analyses for ICU Admission After Colorectal Cancer Surgery

| Characteristics | | β | 95% CI | *P* value |
|---|---|---|---|---|
| (Intercept) | | -2.05 | -2.19 - -1.91 | <.001 |
| Sex | Male | Ref. | | |
| | Female | -0.25 | -0.30 - -0.21 | <.001 |
| Comorbidities | Angina Pectoris | 0.12 | 0.02 - 0.22 | .02 |
| | Myocardial infarction | 0.16 | 0.07 - 0.24 | <.001 |
| | Percutaneous transluminal coronary angioplasty (PTCA) | 0.08 | -0.03 - 0.19 | .13 |
| | Coronary artery bypass grafting (CABG) | 0.04 | -0.07 - 0.14 | .49 |
| | Valvular heart disease | 0.18 | 0.07 - 0.28 | <.001 |
| | Cardiac valve replacement | 0.26 | 0.08 - 0.43 | .005 |
| | Atrial fibrillation/ atrial flutter | 0.26 | 0.19 - 0.33 | <.001 |
| | Cardiac arrhythmia (exclusive atrial fibrillation and flutter) | 0.16 | 0.05 - 0.26 | .003 |
| | Congestive heart failure | 0.32 | 0.20 - 0.45 | <.001 |
| | Cardiomyopathy | 0.50 | 0.31 - 0.70 | <.001 |
| | Medical history of heart transplantation | 0.44 | -1.08 - 1.96 | .56 |
| | Other heart disease | 0.21 | 0.09 - 0.33 | <.001 |
| | Hypertension | 0.03 | -0.02 - 0.08 | .17 |
| | Peripheral vascular disease | 0.09 | -0.03 - 0.20 | .13 |
| | Carotid artery stenosis | 0.06 | -0.20 - 0.33 | .63 |
| | Aortic aneurysm (abdominal/thoracic) | 0.19 | 0.06 - 0.33 | .005 |
| | Other vascular disease | -0.06 | -0.21 - 0.08 | .39 |
| | Non-insulin-dependent diabetes mellitus | 0.10 | 0.03 - 0.16 | .004 |
| | Insulin-dependent diabetes mellitus | 0.11 | 0.00 - 0.22 | .05 |
| | Diabetes mellitus with end-organ damage | 0.15 | -0.14 - 0.43 | .30 |
| | Other diabetes mellitus related comorbidity | -0.20 | -0.61 - 0.20 | .31 |
| | COPD/asthma | 0.27 | 0.21 - 0.33 | <.001 |
| | Pulmonary fibrosis | 0.49 | 0.00 - 0.99 | .05 |
| | History of lung surgery/transplantation | 0.70 | 0.40 - 1.00 | <.001 |
| | Other pulmonary disease | 0.28 | 0.16 - 0.40 | <.001 |
| | Transient ischemic attack | 0.02 | -0.08 - 0.12 | .68 |
| | Cerebrovascular attack | 0.06 | -0.04 - 0.16 | .23 |
| | Myopathy | 0.19 | -0.22 - 0.60 | .36 |
| | Hemiplegia/ paraplegia | 0.65 | 0.24 - 1.06 | .002 |
| | Parkinson's disease/ dementia | 0.02 | -0.15 - 0.19 | .85 |
| | Schizophrenia/ major depressive disorder/ psychosis | 0.06 | -0.11 - 0.23 | .48 |
| | Other neurological disease | -0.10 | -0.22 - 0.01 | .06 |
| | Gastroesophageal reflux disorder / peptic ulcer disease | 0.05 | -0.07 - 0.16 | .44 |
| | Symptomatic cholelithiasis | 0.10 | -0.08 - 0.29 | .27 |
| | Pancreatitis (acute/chronic) | 0.23 | -0.11 - 0.58 | .18 |
| | Inflammatory bowel disease (M. Crohn/ Ulcerative Colitis) | -0.06 | -0.29 - 0.17 | .59 |
| | Diverticulitis | 0.01 | -0.17 - 0.20 | .90 |
| | Liver disease/ failure (cirrhosis/hepatitis) | 0.46 | 0.24 - 0.69 | <.001 |
| | Other gastrointestinal disease | -0.15 | -0.29 - -0.01 | .04 |
| | Chronic kidney disease (creatinine <110) | 0.20 | 0.08 - 0.32 | .001 |
| | Dialysis-dependent kidney failure | 0.82 | 0.28 - 1.37 | .003 |
| | Medical history of renal surgery/ transplantation | 0.28 | 0.03 - 0.54 | .03 |
| | Pregnant at the time of surgery | 0.29 | -1.96 - 2.55 | .80 |
| | Other urogenital disease | -0.19 | -0.29 - -0.09 | <.001 |
| | Deep venous thrombosis | 0.06 | -0.10 - 0.22 | .44 |
| | Pulmonary embolism | 0.10 | -0.08 - 0.27 | .27 |
| | Blood clotting disorder | 0.08 | -0.38 - 0.54 | .74 |
| | Other hematologic disease | 0.06 | -0.25 - 0.37 | .70 |
| | Sarcoidosis/ Besnier Boeck | 0.49 | 0.01 - 0.97 | .05 |

© 2021 van den Bosch T et al. *JAMA Network Open.*

**eTable 7 continued: Multivariate logistic regression analyses for ICU admission after colorectal cancer surgery**

| Characteristics | | β | 95% CI | *P* value |
|---|---|---|---|---|
| | Rheumatoid Arthritis/ SLE/ scleroderma | -0.12 | -0.25 - 0.01 | .06 |
| | Vasculitis | 0.38 | -0.49 - 1.24 | .39 |
| | Other immunodeficiency | -0.05 | -0.16 - 0.06 | .36 |
| | Hypo-/hyperthyroidism | -0.01 | -0.12 - 0.10 | .85 |
| | Hypo-/hyperparathyroidism | 0.04 | -0.32 - 0.40 | .83 |
| Adrenal disease (Addison Disease/ Cushing Syndrome/ Conn syndrome) | | 0.54 | -0.14 - 1.22 | .11 |
| | Other endocrine disease | -0.16 | -0.40 - 0.08 | .18 |
| | HIV/AIDS | -0.98 | -2.21 - 0.26 | .12 |
| | Tuberculosis | 0.27 | -0.10 - 0.64 | .14 |
| | Malaria | 0.53 | -0.28 - 1.34 | .20 |
| | Other infectious disease | -0.04 | -0.36 - 0.27 | .79 |
| | Current untreated malignancy | 0.17 | 0.05 - 0.30 | .006 |
| | Curative malignancy treated <5 years ago | 0.01 | -0.09 - 0.10 | .90 |
| | Curative malignancy treated >5 years ago | 0.09 | 0.01 - 0.18 | .03 |
| | Palliative treated and / or distant metastasis | 0.03 | -0.21 - 0.26 | .84 |
| | Other types of cancer | -0.10 | -0.47 - 0.28 | .62 |
| **Surgical history** | History of esophageal, gastric, pancreatic or spleen surgery | 0.10 | -0.06 - 0.26 | .20 |
| | History of hepatobiliary surgery | 0.02 | -0.07 - 0.11 | .69 |
| | History of bowel surgery | 0.08 | 0.02 - 0.15 | .01 |
| | History of bladder-, prostate-, uterine or ovarian surgery | 0.06 | -0.01 - 0.13 | .11 |
| | Other abdominal surgery in the history | -0.04 | -0.13 - 0.05 | .43 |
| **Stoma before surgical resection** | No stoma | Ref. | | |
| | Ileostomy | 0.35 | -0.05 - 0.74 | .08 |
| | Colostomy | 0.25 | 0.02 - 0.48 | .03 |
| | Type unknown | -0.56 | -1.75 - 0.63 | .35 |
| **Number of tumors found by endoscopy** | 1 | Ref. | | |
| | 2 | -0.04 | -0.70 - 0.62 | .90 |
| | 3+ | 0.17 | -0.54 - 0.88 | .63 |
| **Preoperative MDT** | | -0.12 | -0.19 - -0.05 | .001 |
| **Preoperative surgical procedure** | Stoma | 0.28 | 0.16 - 0.40 | <.001 |
| | Stent | -0.32 | -0.75 - 0.10 | .13 |
| | Metastasectomy | 0.23 | 0.01 - 0.45 | .04 |
| | Appendectomy | 0.40 | -0.04 - 0.83 | .07 |
| | Other | 0.36 | 0.14 - 0.57 | .001 |
| **Neoadjuvant radiotherapy** | No neoadjuvant radiotherapy | Ref. | | |
| | Short course | 0.17 | 0.05 - 0.29 | .005 |
| | Long course without chemotherapy | 0.21 | 0.04 - 0.38 | .02 |
| | Chemoradiation | 0.42 | 0.29 - 0.55 | <.001 |
| **Neoadjuvant chemotherapy** | | 0.07 | -0.02 - 0.17 | <.001 |
| **ASA score** | I | Ref. | | |
| | II | 0.19 | 0.12 - 0.26 | <.001 |
| | III | 0.79 | 0.71 - 0.88 | <.001 |
| | IV | 1.50 | 1.34 - 1.66 | <.001 |
| | V | 1.80 | 0.83 - 2.76 | <.001 |
| **Setting** | Elective | Ref. | | |
| | Elective after placing a stent | 0.56 | 0.29 - 0.84 | <.001 |
| | Urgent | 0.23 | 0.12 - 0.33 | <.001 |
| | Emergency, direct procedure | 0.47 | 0.36 - 0.59 | <.001 |
| **Tumor complications** | No tumor complications | Ref. | | |
| | Fecal peritonitis due to preoperative colorectal perforation | 1.05 | 0.88 - 1.22 | <.001 |
| | Preoperative presence of an abscess | 0.55 | 0.36 - 0.74 | <.001 |
| | Preoperative bowel obstruction/ ileus due to malignancy | 0.19 | 0.10 - 0.28 | <.001 |

© 2021 van den Bosch T et al. *JAMA Network Open.*

## eTable 7 continued: Multivariate logistic regression analyses for ICU admission after colorectal cancer surgery

| Characteristics | | β | 95% CI | *P* value |
|---|---|---|---|---|
| | Preoperative blood loss/ anemia | 0.27 | 0.21 - 0.33 | <.001 |
| | Other preoperative tumor related complications | 0.11 | 0.00 - 0.21 | .05 |
| **Approach** | Open | Ref. | | |
| | Laparoscopic | -0.58 | -0.63 - -0.53 | <.001 |
| | Transanal endoscopic microsurgery (TEM) | -0.39 | -0.95 - 0.18 | .17 |
| | Transanal local excision (open) | 0.02 | -0.71 - 0.75 | .95 |
| | Transanal minimally invasive surgery (TAMIS) | -2.15 | -3.61 - -0.69 | .004 |
| | Transanal total mesorectal excision (TaTME) | -1.29 | -1.79 - -0.79 | <.001 |
| **Metastasis** | Liver metastasis | -0.03 | -0.17 - 0.11 | .65 |
| | Pulmonary metastasis | -0.47 | -0.72 - -0.22 | <.001 |
| | Bone metastasis | 0.10 | -0.69 - 0.90 | .79 |
| | Peritoneal metastasis | 0.65 | 0.420 - 0.88 | <.001 |
| | Ovarian metastasis | 1.54 | 1.00 - 2.09 | <.001 |
| | Brain metastasis | 0.76 | -0.61 - 2.14 | .27 |
| | Other metastasis | 0.06 | -0.21 - 0.33 | .67 |
| **Age** | 60-70 | Ref. | | |
| | <60 | -0.09 | -0.17 - -0.02 | .009 |
| | ≥80 | 0.44 | 0.37 - 0.51 | <.001 |
| | 70-80 | 0.17 | 0.12 - 0.23 | <.001 |
| **BMI** | 18.5-25.0 | Ref. | | |
| | <18.5 | 0.14 | -0.01 - 0.28 | .06 |
| | ≥30.0 | 0.24 | 0.178 - 0.30 | <.001 |
| | 25.0-30.0 | 0.09 | 0.04 - 0.14 | <.001 |
| **Tumor location** | Colon | Ref. | | |
| | Rectum | 0.08 | -0.04 - 0.20 | .18 |
| | Multiple tumors | -0.13 | -0.85 - 0.58 | .71 |
| **Tumor found by screening** | | -0.09 | -0.18 - 0.01 | .07 |
| **Procedure** | (low) Anterior/ sigmoid resection | Ref. | | |
| | Ileocecal resection | -0.47 | -0.72 - -0.22 | <.001 |
| | (extended) Right hemicolectomy | -0.16 | -0.22 - -0.10 | <.001 |
| | Local excision followed by a total mesorectal excision | 0.22 | -0.46 - 0.89 | .53 |
| | Same procedure for multiple tumors | 0.16 | -0.20 - 0.52 | .38 |
| | Two different procedures of which at least 1 procedure for rectal cancer | 0.54 | 0.08 - 1.00 | .02 |
| | Two different colon procedures | 0.34 | -0.04 - 0.71 | .07 |
| | Transversectomy | -0.07 | -0.23 - 0.10 | .43 |
| | (extended) Left hemicolectomy | 0.15 | 0.07 - 0.24 | <.001 |
| | Subtotal colectomy | 0.71 | 0.53 - 0.88 | <.001 |
| | Abdominoperineal resection | 0.32 | 0.24 - 0.41 | <.001 |
| | Panproctocolectomy | -0.49 | -1.01 - 0.04 | .07 |
| | Other | -0.27 | -0.53 – 0.00 | .05 |
| | Local excision | -14.04 | -115.48 - 87.40 | .78 |
| | Local excision followed by an abdominoperineal resection | 0.14 | -0.32 - 0.60 | .54 |
| **M-stage** | M0 | Ref. | | |
| | M1 | 0.15 | 0.07 - 0.24 | <.001 |
| **T-stage** | T1 | Ref. | | |
| | T2 | 0.05 | -0.05 - 0.15 | .30 |
| | T3 | 0.02 | -0.07 - 0.12 | .60 |
| | T4 | 0.30 | 0.19 - 0.40 | <.001 |
| | T0 | 0.30 | -0.15 - 0.74 | .19 |

**eTable 7:** Logistic regression coefficients for ICU admission on 62,501 patient records. The *P*-values are given as calculated by the Wald test. When no reference level is reported, variables are binary and the reference level is absence of that variable.

© 2021 van den Bosch T et al. *JAMA Network Open.*

**eTable 8.** Multivariate Logistic Regression Analyses for Readmission After Colorectal Cancer Surgery

| Characteristics | | β | 95% CI | *P-value* |
|---|---|---|---|---|
| **(Intercept)** | | -3.77 | -4.03 - -3.52 | <.001 |
| **Sex** | Male | Ref. | | |
| | Female | -0.10 | -0.17 - -0.03 | .007 |
| **Comorbidities** | Angina Pectoris | 0.06 | -0.09 - 0.21 | .44 |
| | Myocardial infarction | 0.01 | -0.12 - 0.15 | .84 |
| | Percutaneous transluminal coronary angioplasty (PTCA) | 0.12 | -0.04 - 0.27 | .15 |
| | Coronary artery bypass grafting (CABG) | 0.07 | -0.09 - 0.23 | .38 |
| | Valvular heart disease | 0.05 | -0.11 - 0.22 | .52 |
| | Cardiac valve replacement | 0.28 | 0.02 - 0.55 | .04 |
| | Atrial fibrillation/ atrial flutter | 0.14 | 0.03 - 0.25 | .02 |
| | Cardiac arrhythmia (exclusive atrial fibrillation and flutter) | -0.05 | -0.21 - 0.12 | .56 |
| | Congestive heart failure | -0.21 | -0.44 - 0.03 | .08 |
| | Cardiomyopathy | 0.09 | -0.23 - 0.40 | .60 |
| | Medical history of heart transplantation | -11.08 | -346.08 - 323.92 | .95 |
| | Other heart disease | 0.12 | -0.06 - 0.30 | .18 |
| | Hypertension | 0.04 | -0.03 - 0.11 | .29 |
| | Peripheral vascular disease | -0.06 | -0.24 - 0.13 | .55 |
| | Carotid artery stenosis | 0.29 | -0.08 - 0.67 | .12 |
| | Aortic aneurysm (abdominal/thoracic) | 0.26 | 0.06 - 0.46 | .009 |
| | Other vascular disease | 0.08 | -0.13 - 0.29 | .44 |
| | Non-insulin-dependent diabetes mellitus | 0.07 | -0.03 - 0.17 | .17 |
| | Insulin-dependent diabetes mellitus | 0.06 | -0.10 - 0.22 | .47 |
| | Diabetes mellitus with end-organ damage | -0.10 | -0.56 - 0.36 | .66 |
| | Other diabetes mellitus related comorbidity | 0.19 | -0.34 - 0.72 | .48 |
| | COPD/asthma | 0.00 | -0.10 - 0.10 | 1.00 |
| | Pulmonary fibrosis | 0.05 | -0.79 - 0.90 | .90 |
| | History of lung surgery/transplantation | -0.02 | -0.52 - 0.48 | .93 |
| | Other pulmonary disease | -0.02 | -0.22 - 0.18 | .84 |
| | Transient ischemic attack | 0.04 | -0.12 - 0.19 | .63 |
| | Cerebrovascular attack | 0.20 | 0.06 - 0.35 | .006 |
| | Myopathy | -0.20 | -0.89 - 0.49 | .56 |
| | Hemiplegia/ paraplegia | -0.11 | -0.81 - 0.60 | .77 |
| | Parkinson’s disease/ dementia | -0.02 | -0.29 - 0.26 | .91 |
| | Schizophrenia/ major depressive disorder/ psychosis | 0.21 | -0.03 - 0.44 | .08 |
| | Other neurological disease | -0.09 | -0.25 - 0.08 | .29 |
| | Gastroesophageal reflux disorder / peptic ulcer disease | 0.07 | -0.11 - 0.24 | .44 |
| | Symptomatic cholelithiasis | 0.23 | -0.02 - 0.49 | .07 |
| | Pancreatitis (acute/chronic) | 0.27 | -0.21 - 0.75 | .26 |
| | Inflammatory bowel disease (M. Crohn/ Ulcerative Colitis) | 0.30 | 0.01 - 0.59 | .04 |
| | Diverticulitis | -0.04 | -0.33 - 0.25 | .76 |
| | Liver disease/ failure (cirrhosis/hepatitis) | 0.25 | -0.07 - 0.58 | .13 |
| | Other gastrointestinal disease | 0.10 | -0.10 - 0.29 | .32 |
| | Chronic kidney disease (creatinine <110) | 0.18 | -0.01 - 0.36 | .06 |
| | Dialysis-dependent kidney failure | 0.63 | -0.08 - 1.33 | .08 |
| | Medical history of renal surgery/ transplantation | 0.11 | -0.27 - 0.483 | .58 |
| | Pregnant at the time of surgery | -10.91 | -479.61 - 457.78 | .96 |
| | Other urogenital disease | 0.02 | -0.13 - 0.17 | .77 |
| | Deep venous thrombosis | 0.06 | -0.16 - 0.29 | .58 |
| | Pulmonary embolism | 0.32 | 0.09 - 0.56 | .007 |
| | Blood clotting disorder | -0.80 | -1.81 - 0.22 | .12 |

© 2021 van den Bosch T et al. *JAMA Network Open.*

## eTable 8 continued : Multivariate logistic regression analyses for readmission after colorectal cancer surgery

| Characteristics | | β | 95% CI | *P* value |
|---|---|---|---|---|
| | Other hematologic disease | -0.38 | -0.91 - 0.16 | .16 |
| | Sarcoidosis/ Besnier Boeck | 0.15 | -0.56 - 0.85 | .68 |
| | Rheumatoid Arthritis/ SLE/ scleroderma | 0.05 | -0.14 - 0.23 | .62 |
| | Vasculitis | -0.03 | -1.51 - 1.44 | .97 |
| | Other immunodeficiency | 0.03 | -0.12 - 0.19 | .67 |
| | Hypo-/hyperthyroidism | 0.06 | -0.10 - 0.22 | .45 |
| | Hypo-/hyperparathyroidism | 0.46 | 0.02 - 0.91 | .04 |
| Adrenal disease (Addison Disease/ Cushing Syndrome/ Conn syndrome) | | -0.08 | -1.12 - 0.97 | .89 |
| | Other endocrine disease | 0.26 | -0.04 - 0.56 | .09 |
| | HIV/AIDS | -0.34 | -1.81 - 1.13 | .65 |
| | Tuberculosis | 0.27 | -0.26 - 0.80 | .32 |
| | Malaria | -0.01 | -1.48 - 1.46 | .99 |
| | Other infectious disease | -0.36 | -0.89 - 0.18 | .18 |
| | Current untreated malignancy | 0.13 | -0.06 - 0.32 | .17 |
| | Curative malignancy treated <5 years ago | 0.01 | -0.14 - 0.15 | .93 |
| | Curative malignancy treated >5 years ago | 0.12 | -0.01 - 0.24 | .06 |
| | Palliative treated and / or distant metastasis | -0.43 | -0.87 - 0.01 | .05 |
| | Other types of cancer | 0.20 | -0.34 - 0.73 | .46 |
| **Surgical history** | History of esophageal, gastric, pancreatic or spleen surgery | 0.06 | -0.18 - 0.30 | .62 |
| | History of hepatobiliary surgery | 0.01 | -0.13 - 0.14 | .94 |
| | History of bowel surgery | 0.17 | 0.08 - 0.26 | <.001 |
| | History of bladder-, prostate-, uterine or ovarian surgery | 0.07 | -0.04 - 0.17 | .21 |
| | Other abdominal surgery in the history | -0.06 | -0.20 - 0.07 | .35 |
| **Stoma before surgical resection** | No stoma | Ref. | | |
| | Ileostomy | 0.40 | -0.11 - 0.91 | .12 |
| | Colostomy | 0.37 | 0.06 - 0.68 | .02 |
| | Type unknown | 0.91 | -0.35 - 2.18 | .15 |
| **Number of tumors found by endoscopy** | 1 | Ref. | | |
| | 2 | -1.70 | -3.73 - 0.34 | .10 |
| | 3+ | -1.48 | -3.54 - 0.58 | .16 |
| **Preoperative MDT** | | 0.24 | 0.11 - 0.36 | <.001 |
| **Preoperative surgical procedure** | Stoma | -0.02 | -0.20 - 0.16 | .83 |
| | Stent | -0.01 | -0.72 - 0.70 | .98 |
| | Metastasectomy | -0.11 | -0.41 - 0.20 | .49 |
| | Appendectomy | -0.19 | -0.89 - 0.50 | .58 |
| | Other | -0.05 | -0.39 - 0.28 | .75 |
| **Neoadjuvant radiotherapy** | No Neoadjuvant radiotherapy | Ref. | | |
| | Short course | 0.11 | -0.04 - 0.25 | .13 |
| | Long course without chemotherapy | -0.03 | -0.26 - 0.20 | .81 |
| | Chemoradiation | 0.31 | 0.15 - 0.48 | <.001 |
| **Neoadjuvant chemotherapy** | | 0.02 | -0.11 - 0.14 | .13 |
| **ASA score** | I | Ref. | | |
| | II | 0.19 | 0.09 - 0.28 | <.001 |
| | III | 0.40 | 0.28 - 0.52 | <.001 |
| | IV | 0.01 | -0.29 - 0.31 | .94 |
| | V | -10.67 | -214.24 - 192.90 | .92 |
| **Setting** | Elective | Ref. | | |
| | Elective after placing a stent | -0.04 | -0.50 - 0.42 | .85 |
| | Urgent | 0.18 | 0.01 - 0.36 | .04 |
| | Emergency, direct procedure | 0.19 | -0.02 - 0.39 | .08 |

© 2021 van den Bosch T et al. *JAMA Network Open.*

## eTable 8 continued : Multivariate logistic regression analyses for readmission after colorectal cancer surgery

| Characteristics | | β | 95% CI | *P* value |
|---|---|---|---|---|
| **Tumor complications** | Fecal peritonitis due to preoperative colorectal perforation | 0.00 | -0.31 - 0.31 | .99 |
| | Preoperative presence of an abscess | -0.13 | -0.48 - 0.22 | .46 |
| | Preoperative bowel obstruction/ ileus due to malignancy | 0.09 | -0.06 - 0.24 | .22 |
| | Preoperative blood loss/ anemia | 0.12 | 0.04 - 0.21 | .003 |
| | Other preoperative tumor related complications | 0.16 | 0.02 - 0.31 | .03 |
| **Approach** | Open | Ref. | | |
| | Laparoscopic | 0.13 | 0.05 - 0.20 | .001 |
| | Transanal endoscopic microsurgery (TEM) | 0.40 | -0.24 - 1.05 | .21 |
| | Transanal local excision (open) | 0.34 | -0.56 - 1.24 | .45 |
| | Transanal minimally invasive surgery (TAMIS) | -0.69 | -1.80 - 0.41 | .21 |
| | Transanal total mesorectal excision (TaTME) | 0.52 | 0.10 - 0.93 | .01 |
| **Surgical conversion** | No conversion | Ref. | | |
| | Early | 0.23 | -0.56 - 1.03 | .56 |
| | Late | 0.22 | -0.59 - 1.02 | .59 |
| **Reason for surgical conversion** | No conversion | Ref. | | |
| | Complexity | 0.08 | -0.74 - 0.90 | .85 |
| | Accessibility | 0.01 | -0.78 - 0.81 | .97 |
| | Preoperative tumor complication | 0.05 | -0.84 - 0.94 | .91 |
| **Intraoperative complications** | Blood loss for which blood transfusion was required | -0.21 | -0.62 - 0.20 | .32 |
| | Damage of the spleen for which a splenectomy was required | 0.41 | -0.35 - 1.16 | .29 |
| | Damage to the pancreas, liver, d. choledochus, gallbladder | 0.96 | 0.12 - 1.80 | .02 |
| | Damage to the intestine | 0.52 | 0.25 - 0.80 | <.001 |
| | Damage to the ureter/urethra | 0.36 | -0.07 - 0.79 | .10 |
| | Damage to the urine bladder | 0.27 | -0.38 - 0.92 | .41 |
| | Damage to the vagina | 0.41 | -0.55 - 1.38 | .40 |
| | Perforation intra-abdominal during a transanal local excision | 1.84 | 0.82 - 2.86 | <.001 |
| **Primary anastomosis** | | -0.08 | -0.24 - 0.08 | .31 |
| **Stoma** | No stoma | Ref. | | |
| | Loop ileostomy | 0.55 | 0.44 - 0.66 | <.001 |
| | End ileostomy | 0.49 | 0.26 - 0.73 | <.001 |
| | Loop colostomy | -0.03 | -0.27 - 0.22 | .83 |
| | End colostomy | 0.05 | -0.13 - 0.22 | .59 |
| | Stoma of unknown type | -1.96 | -3.97 - 0.06 | .05 |
| **Radio- or chemo therapy during surgery** | No | Ref. | | |
| | IORTC | 0.09 | -0.45 - 0.63 | .74 |
| | HIPEC | 0.97 | 0.41 - 1.53 | <.001 |
| **Additional resection for local ingrowth** | No | Ref. | | |
| | Extensive | 0.14 | -0.03 - 0.31 | .11 |
| | Limited | 0.02 | -0.13 - 0.17 | .78 |
| **Specification additional resection** | Resection of the omentum | -0.19 | -0.58 - 0.21 | .35 |
| | Resection of the liver. or RFA | 0.23 | -0.04 - 0.49 | .09 |
| | Resection of the lung | -0.26 | -1.75 - 1.23 | .73 |
| | Resection of peritoneal metastases | 0.13 | -0.33 - 0.58 | .58 |
| | Lymph node dissection | -0.06 | -0.50 - 0.39 | .80 |
| | Resection of other abdominal organs | -0.22 | -0.62 - 0.19 | .29 |
| | Other abdominal resection | 0.11 | -0.05 - 0.27 | .17 |
| **Metastasis** | Liver metastasis | 0.16 | -0.05 - 0.37 | .12 |

© 2021 van den Bosch T et al. *JAMA Network Open.*

## eTable 8 continued : Multivariate logistic regression analyses for readmission after colorectal cancer surgery

| Characteristics | | β | 95% CI | *P* value |
|---|---|---|---|---|
| | Pulmonary metastasis | -0.21 | -0.58 - 0.16 | .25 |
| | Bone metastasis | 0.53 | -0.55 - 1.62 | .33 |
| | Peritoneal metastasis | 0.02 | -0.37 - 0.42 | .91 |
| | Ovarian metastasis | 0.90 | 0.16 - 1.63 | .02 |
| | Brain metastasis | -11.6 | -339.35 - 316.19 | .94 |
| | Other metastasis | -0.03 | -0.45 - 0.38 | .88 |
| **Age** | 60-70 | Ref. | | |
| | <60 | 0.11 | 0.02 - 0.20 | .02 |
| | ≥80 | -0.26 | -0.37 - -0.15 | <.001 |
| | 70-80 | -0.02 | -0.10 - 0.06 | .56 |
| **BMI** | 18.5-25.0 | Ref. | | |
| | <18.5 | -0.21 | -0.45 - 0.02 | .07 |
| | ≥30.0 | 0.05 | -0.04 - 0.14 | .24 |
| | 25.0-30.0 | 0.04 | -0.03 - 0.11 | .24 |
| **Tumor location** | Colon | Ref. | | |
| | Rectum | 0.52 | 0.37 - 0.67 | <.001 |
| | Multiple tumors | 2.00 | -0.08 - 4.08 | .06 |
| **Tumor found by screening** | | 0.01 | -0.11 - 0.13 | .85 |
| **Procedure** | (low) Anterior/ sigmoid resection | Ref. | | |
| | Ileocecal resection | 0.37 | 0.01 - 0.73 | .04 |
| | (extended) Right hemicolectomy | 0.24 | 0.14 - 0.34 | <.001 |
| | Local excision followed by a total mesorectal excision | -0.08 | -0.98 - 0.82 | .86 |
| | Same procedure for multiple tumors | 0.06 | -0.47 - 0.59 | .82 |
| | Two different procedures of which at least 1 procedure for rectal cancer | 0.23 | -0.43 - 0.89 | .48 |
| | Two different colon procedures | 0.18 | -0.37 - 0.72 | .53 |
| | Transversectommy | 0.23 | -0.04 - 0.50 | .10 |
| | (extended) Left hemicolectomy | 0.40 | 0.27 - 0.54 | <.001 |
| | Subtotal colectomy | 0.31 | 0.02 - 0.59 | .03 |
| | Abdominoperineal resection | 0.04 | -0.10 - 0.18 | .58 |
| | Panproctocolectomy | -0.13 | -0.77 - 0.50 | .68 |
| | Other | -0.13 | -0.55 - 0.30 | .56 |
| | Local excision | -0.27 | -0.94 - 0.41 | .43 |
| | Local excision followed by an abdominal perineal resection | -1.00 | -1.82 - -0.18 | .02 |
| **M-stage** | M0 | Ref. | | |
| | M1 | 0.03 | -0.11 - 0.18 | .64 |
| **T-stage** | T1 | Ref. | | |
| | T2 | 0.12 | -0.02 - 0.26 | .09 |
| | T3 | 0.11 | -0.02 - 0.24 | .10 |
| | T4 | 0.16 | 0.00 - 0.32 | .05 |
| | T0 | 0.41 | -0.21 - 1.03 | .19 |

**eTable 8:** Logistic regression coefficients for readmission on 62,501 patient records. The *P*-values are given as calculated by the Wald test. When no reference level is reported, variables are binary and the reference level is absence of that variable.

© 2021 van den Bosch T et al. *JAMA Network Open.*

**eTable 9.** Multivariate Logistic Regression Analyses for Prolonged Stay in Hospital After Colorectal Cancer Surgery

| Characteristics | | β | 95% CI | *P* value |
|---|---|---|---|---|
| **(Intercept)** | | -3.16 | -3.41 - -2.92 | <.001 |
| **Sex** | Male | Ref. | | |
| | Female | -0.27 | -0.34 - -0.20 | <.001 |
| **Comorbidities** | Angina Pectoris | -0.07 | -0.21 - 0.08 | .36 |
| | Myocardial infarction | 0.03 | -0.09 - 0.16 | .60 |
| | Percutaneous transluminal coronary angioplasty (PTCA) | 0.01 | -0.15 - 0.16 | .91 |
| | Coronary artery bypass grafting (CABG) | -0.17 | -0.33 - -0.01 | .04 |
| | Valvular heart disease | -0.04 | -0.19 - 0.12 | .62 |
| | Cardiac valve replacement | 0.11 | -0.15 - 0.37 | .41 |
| | Atrial fibrillation/ atrial flutter | 0.02 | -0.09 - 0.13 | .71 |
| | Cardiac arrhythmia (exclusive atrial fibrillation and flutter) | 0.01 | -0.14 - 0.16 | .93 |
| | Congestive heart failure | 0.24 | 0.07 - 0.41 | .006 |
| | Cardiomyopathy | 0.06 | -0.22 - 0.35 | .66 |
| | Medical history of heart transplantation | 1.01 | -0.67 - 2.69 | .23 |
| | Other heart disease | -0.03 | -0.21 - 0.14 | .70 |
| | Hypertension | -0.06 | -0.13 - 0.01 | .07 |
| | Peripheral vascular disease | 0.13 | -0.03 - 0.29 | .12 |
| | Carotid artery stenosis | -0.04 | -0.42 - 0.34 | .83 |
| | Aortic aneurysm (abdominal/thoracic) | 0.12 | -0.07 - 0.30 | .22 |
| | Other vascular disease | 0.05 | -0.15 - 0.25 | .61 |
| | Non-insulin-dependent diabetes mellitus | 0.03 | -0.06 - 0.12 | .52 |
| | Insulin-dependent diabetes mellitus | 0.09 | -0.06 - 0.25 | .22 |
| | Diabetes mellitus with end-organ damage | -0.27 | -0.71 - 0.17 | .23 |
| | Other diabetes mellitus related comorbidity | 0.04 | -0.50 - 0.57 | .89 |
| | COPD/asthma | 0.21 | 0.12 - 0.30 | <.001 |
| | Pulmonary fibrosis | 0.71 | 0.11 - 1.30 | .02 |
| | History of lung surgery/transplantation | -0.09 | -0.56 - 0.37 | .69 |
| | Other pulmonary disease | -0.13 | -0.31 - 0.06 | .18 |
| | Transient ischemic attack | 0.00 | -0.14 - 0.15 | .98 |
| | Cerebrovascular attack | 0.06 | -0.08 - 0.19 | .43 |
| | Myopathy | 0.42 | -0.11 - 0.95 | .11 |
| | Hemiplegia/ paraplegia | 0.39 | -0.15 - 0.94 | .15 |
| | Parkinson’s disease/ dementia | 0.15 | -0.07 - 0.37 | .19 |
| | Schizophrenia/ major depressive disorder/ psychosis | 0.04 | -0.20 - 0.28 | .74 |
| | Other neurological disease | 0.16 | 0.01 - 0.30 | .03 |
| | Gastroesophageal reflux disorder / peptic ulcer disease | 0.03 | -0.14 - 0.19 | .75 |
| | Symptomatic cholelithiasis | 0.05 | -0.20 - 0.30 | .71 |
| | Pancreatitis (acute/chronic) | 0.43 | -0.00 - 0.87 | .05 |
| | Inflammatory bowel disease (M. Crohn/ Ulcerative Colitis) | -0.17 | -0.50 - 0.16 | .31 |
| | Diverticulitis | -0.06 | -0.32 - 0.19 | .63 |
| | Liver disease/ failure (cirrhosis/hepatitis) | 0.19 | -0.13 - 0.50 | .25 |
| | Other gastrointestinal disease | 0.15 | -0.04 - 0.33 | .11 |
| | Chronic kidney disease (creatinine <110) | 0.08 | -0.09 - 0.25 | .33 |
| | Dialysis-dependent kidney failure | 0.55 | -0.11 - 1.20 | .10 |
| | Medical history of renal surgery/ transplantation | -0.08 | -0.47 - 0.30 | .67 |
| | Pregnant at the time of surgery | -9.06 | -179.73 - 161.62 | .92 |
| | Other urogenital disease | 0.02 | -0.12 - 0.15 | .82 |
| | Deep venous thrombosis | 0.17 | -0.04 - 0.38 | .10 |
| | Pulmonary embolism | 0.03 | -0.21 - 0.27 | .79 |
| | Blood clotting disorder | -0.53 | -1.33 - 0.27 | .19 |
| | Other hematologic disease | 0.16 | -0.25 - 0.58 | .43 |
| | Sarcoidosis/ Besnier Boeck | 0.03 | -0.71 - 0.78 | .93 |

© 2021 van den Bosch T et al. *JAMA Network Open.*

## eTable 9 continued: Multivariate logistic regression analyses for prolonged stay in hospital after colorectal cancer surgery

| Characteristics | | β | 95% CI | *P* value |
|---|---|---|---|---|
| | Rheumatoid Arthritis/ SLE/ scleroderma | 0.08 | -0.09 - 0.25 | .35 |
| | Vasculitis | 0.02 | -1.23 - 1.27 | .98 |
| | Other immunodeficiency | 0.21 | 0.07 - 0.34 | .003 |
| | Hypo-/hyperthyroidism | -0.02 | -0.17 - 0.14 | .82 |
| | Hypo-/hyperparathyroidism | -0.07 | -0.59 - 0.45 | .79 |
| Adrenal disease (Addison Disease/ Cushing Syndrome/ Conn syndrome) | | 0.32 | -0.64 - 1.27 | .51 |
| | Other endocrine disease | 0.07 | -0.24 - 0.38 | .65 |
| | HIV/AIDS | 0.45 | -0.67 - 1.56 | .43 |
| | Tuberculosis | 0.15 | -0.37 - 0.68 | .56 |
| | Malaria | -0.54 | -2.02 - 0.93 | .47 |
| | Other infectious disease | 0.45 | 0.08 - 0.82 | .02 |
| | Current untreated malignancy | 0.15 | -0.03 - 0.32 | .09 |
| | Curative malignancy treated <5 years ago | 0.07 | -0.07 - 0.20 | .31 |
| | Curative malignancy treated >5 years ago | 0.09 | -0.02 - 0.21 | .11 |
| | Palliative treated and / or distant metastasis | -0.02 | -0.35 - 0.31 | .92 |
| | Other types of cancer | -0.44 | -1.02 - 0.14 | .13 |
| **Surgical history** | History of esophageal, gastric, pancreatic or spleen surgery | 0.13 | -0.09 - 0.34 | .24 |
| | History of hepatobiliary surgery | 0.16 | 0.03 - 0.28 | .01 |
| | History of bowel surgery | 0.05 | -0.04 - 0.14 | .29 |
| | History of bladder-, prostate-, uterine or ovarian surgery | 0.03 | -0.08 - 0.12 | .62 |
| | Other abdominal surgery in the history | -0.05 | -0.18 - 0.08 | .42 |
| **Stoma before surgical resection** | No stoma | Ref. | | |
| | Ileostomy | 0.40 | -0.12 - 0.91 | .13 |
| | Colostomy | -0.23 | -0.59 - 0.12 | .19 |
| | Type unknown | 0.65 | -0.49 - 1.80 | .26 |
| **Number of tumors found by endoscopy** | 1 | Ref. | | |
| | 2 | 0.67 | -0.06 - 1.41 | .07 |
| | 3+ | 0.53 | -0.29 - 1.34 | .20 |
| **Preoperative MDT** | | -0.16 | -0.26 - -0.06 | .002 |
| **Preoperative surgical procedure** | Stoma | 0.02 | -0.16 - 0.20 | .83 |
| | Stent | 0.67 | 0.13 - 1.21 | .01 |
| | Metastasectomy | -0.26 | -0.60 - 0.08 | .13 |
| | Appendectomy | -0.45 | -1.23 - 0.33 | .25 |
| | Other | 0.18 | -0.14 - 0.48 | .25 |
| **Neoadjuvant radiotherapy** | No neoadjuvant radiotherapy | Ref. | | |
| | Short course | 0.27 | 0.12 - 0.42 | <.001 |
| | Long course without chemotherapy | 0.12 | -0.11 - 0.35 | .31 |
| | Chemoradiation | -0.08 | -0.25 - 0.10 | .37 |
| **Neoadjuvant chemotherapy** | | 0.17 | 0.04 - 0.31 | .01 |
| **ASA score** | I | Ref. | | |
| | II | 0.23 | 0.13 - 0.33 | <.001 |
| | III | 0.60 | 0.48 - 0.72 | <.001 |
| | IV | 0.79 | 0.57 - 1.00 | <.001 |
| | V | -0.43 | -1.73 - 0.86 | .51 |
| **Setting** | Elective | Ref. | | |
| | Elective after placing a stent | -0.43 | -0.91 - 0.05 | .08 |
| | Urgent | 0.34 | 0.20 - 0.49 | <.001 |
| | Emergency, direct procedure | 0.27 | 0.11 - 0.44 | <.001 |
| **Tumor complications** | Fecal peritonitis due to preoperative colorectal perforation | 0.61 | 0.40 - 0.82 | <.001 |
| | Preoperative presence of an abscess | 0.30 | 0.04 - 0.55 | .02 |
| | Preoperative bowel obstruction/ ileus due to malignancy | -0.03 | -0.16 - 0.10 | .61 |

© 2021 van den Bosch T et al. *JAMA Network Open.*

**eTable 9 continued: Multivariate logistic regression analyses for prolonged stay in hospital after colorectal cancer surgery**

| Characteristics | | β | 95% CI | *P* value |
|---|---|---|---|---|
| | Preoperative blood loss/ anemia | 0.02 | -0.06 - 0.10 | .61 |
| | Other preoperative tumor related complications | 0.22 | 0.08 - 0.36 | .001 |
| **Approach** | Open | Ref. | | |
| | Laparoscopic | -0.47 | -0.55 - -0.40 | <.001 |
| | Transanal endoscopic microsurgery (TEM) | 0.14 | -0.41 - 0.69 | .61 |
| | Transanal local excision (open) | -0.12 | -1.06 - 0.83 | .80 |
| | Transanal minimally invasive surgery (TAMIS) | 0.33 | -0.38 - 1.04 | .36 |
| | Transanal total mesorectal excision (TaTME) | -0.36 | -0.86 - 0.14 | .16 |
| **Surgical conversion** | No conversion | Ref. | | |
| | Early | 0.64 | -0.05 - 1.33 | .07 |
| | Late | 0.95 | 0.25 - 1.64 | .007 |
| **Reason for surgical conversion** | No conversion | Ref. | | |
| | Complexity | -0.23 | -0.94 - 0.48 | .52 |
| | Accessibility | -0.18 | -0.87 - 0.51 | .61 |
| | Preoperative tumor complications | -0.15 | -0.93 - 0.62 | .70 |
| **Intraoperative complications** | Blood loss for which blood transfusion was required | 0.15 | -0.18 - 0.48 | .38 |
| | Damage of the spleen for which a splenectomy was required | 0.37 | -0.29 - 1.03 | .27 |
| | Damage to the pancreas, liver, d. choledochus, gallbladder | 1.19 | 0.44 - 1.94 | .002 |
| | Damage to the intestine | 0.39 | 0.12 - 0.65 | .004 |
| | Damage to the ureter/urethra | 0.67 | 0.28 - 1.05 | <.001 |
| | Damage to the urine bladder | 0.39 | -0.22 - 1.00 | .21 |
| | Damage to the vagina | 1.01 | 0.20 - 1.83 | .01 |
| | Perforation intra-abdominal during a transanal local excision | 0.77 | -0.38 - 1.92 | .18 |
| **Primary anastomosis** | | 0.04 | -0.12 - 0.19 | .65 |
| **Stoma** | No stoma | Ref. | | |
| | Loop ileostomy | 0.69 | 0.58 - 0.81 | <.001 |
| | End ileostomy | 0.19 | -0.04 - 0.41 | .10 |
| | Loop colostomy | 0.21 | -0.01 - 0.43 | .05 |
| | End colostomy | 0.21 | 0.04 - 0.38 | .01 |
| | Stoma of unknown type | -0.07 | -0.86 - 0.73 | .87 |
| **Radio- or chemo-therapy during surgery** | No | Ref. | | |
| | IORTC | -0.21 | -0.82 - 0.39 | .49 |
| | HIPEC | 0.92 | 0.41 - 1.42 | <.001 |
| **Additional resection for local ingrowth** | No additional resection | Ref. | | |
| | Extensive | 0.45 | 0.31 - 0.59 | <.001 |
| | Limited | 0.05 | -0.08 - 0.19 | .43 |
| **Specification additional resection** | Resection of the omentum | -0.06 | -0.38 - 0.27 | .74 |
| | Resection of the liver. or RFA | 0.45 | 0.21 - 0.70 | <.001 |
| | Resection of the lung | 0.14 | -1.36 - 1.64 | .85 |
| | Resection of peritoneal metastases | -0.04 | -0.43 - 0.35 | .84 |
| | Lymph node dissection | 0.08 | -0.31 - 0.48 | .67 |
| | Resection of other abdominal organs | 0.16 | -0.16 - 0.47 | .33 |
| | Other abdominal resection | 0.20 | 0.06 - 0.34 | .004 |
| **Metastasis** | Liver metastasis | -0.11 | -0.32 - 0.09 | .28 |
| | Pulmonary metastasis | -0.56 | -0.97 - -0.16 | .006 |
| | Bone metastasis | -0.70 | -2.18 - 0.77 | .34 |
| | Peritoneal metastasis | -0.41 | -0.80 - -0.01 | .04 |
| | Ovarian metastasis | 0.35 | -0.43 - 1.13 | .37 |

© 2021 van den Bosch T et al. *JAMA Network Open.*

## eTable 9 continued: Multivariate logistic regression analyses for prolonged stay in hospital after colorectal cancer surgery

| Characteristics | | β | 95% CI | *P* value |
|---|---|---|---|---|
| | Brain metastasis | -0.01 | -2.17 - 2.15 | .99 |
| | Other metastasis | 0.24 | -0.12 - 0.61 | .18 |
| **Age** | 60-70 | Ref. | | |
| | <60 | -0.20 | -0.30 - -0.10 | <.001 |
| | ≥80 | 0.33 | 0.23 - 0.43 | <.001 |
| | 70-80 | 0.22 | 0.14 - 0.30 | <.001 |
| **BMI** | 18.5-25.0 | Ref. | | |
| | <18.5 | 0.13 | -0.06 - 0.32 | .18 |
| | ≥30.0 | 0.11 | 0.03 - 0.20 | .01 |
| | 25.0-30.0 | -0.01 | -0.08 - 0.06 | .73 |
| **Tumor location** | Colon | Ref. | | |
| | Rectum | 0.29 | 0.13 - 0.44 | <.001 |
| | Multiple tumors | -0.31 | -1.12 - 0.51 | .45 |
| **Tumor found by screening** | | -0.34 | -0.49 - -0.20 | <.001 |
| **Procedure** | (low) Anterior/sigmoid resection | Ref. | | |
| | Ileocecal resection | 0.02 | -0.32 - 0.35 | .93 |
| | (extended) Right hemicolectomy | 0.14 | 0.05 - 0.24 | .003 |
| | Local excision followed by a total mesorectal excision | 1.29 | 0.66 - 1.91 | <.001 |
| | Same procedure for multiple tumors | 0.02 | -0.42 - 0.46 | .93 |
| | Two different procedures of which at least 1 procedure for rectal cancer | 0.04 | -0.55 - 0.64 | .88 |
| | Two different colon procedures | 0.21 | -0.24 - 0.66 | .36 |
| | Transversectomy | 0.18 | -0.05 - 0.41 | .13 |
| | (extended) Left hemicolectomy | 0.26 | 0.14 - 0.39 | <.001 |
| | Subtotal colectomy | 0.79 | 0.55 - 1.02 | <.001 |
| | Abdominoperineal resection | 0.05 | -0.09 - 0.19 | .50 |
| | Panproctocolectomy | 0.65 | 0.12 - 1.17 | .01 |
| | Other | 0.21 | -0.12 - 0.53 | .21 |
| | Local excision | -2.28 | -3.11 - -1.45 | <.001 |
| | Local excision followed by an abdominoperineal resection | 1.05 | 0.61 - 1.48 | <.001 |
| **M-stage** | M0 | Ref. | | |
| | M1 | 0.00 | -0.12 - 0.13 | .95 |
| **T-stage** | T1 | Ref. | | |
| | T2 | 0.02 | -0.12 - 0.16 | .77 |
| | T3 | 0.10 | -0.03 - 0.24 | .14 |
| | T4 | 0.18 | 0.03 - 0.34 | .02 |
| | T0 | 0.53 | -0.08 - 1.14 | .08 |

**eTable 9:** Logistic regression coefficients for prolonged stay in hospital on 62,501 patient records. The *P*-values are given as calculated by the Wald test. When no reference level is reported, variables are binary and the reference level is absence of that variable.

© 2021 van den Bosch T et al. *JAMA Network Open.*

**eFigure 1.** Pairwise AUC Comparisons for all Models

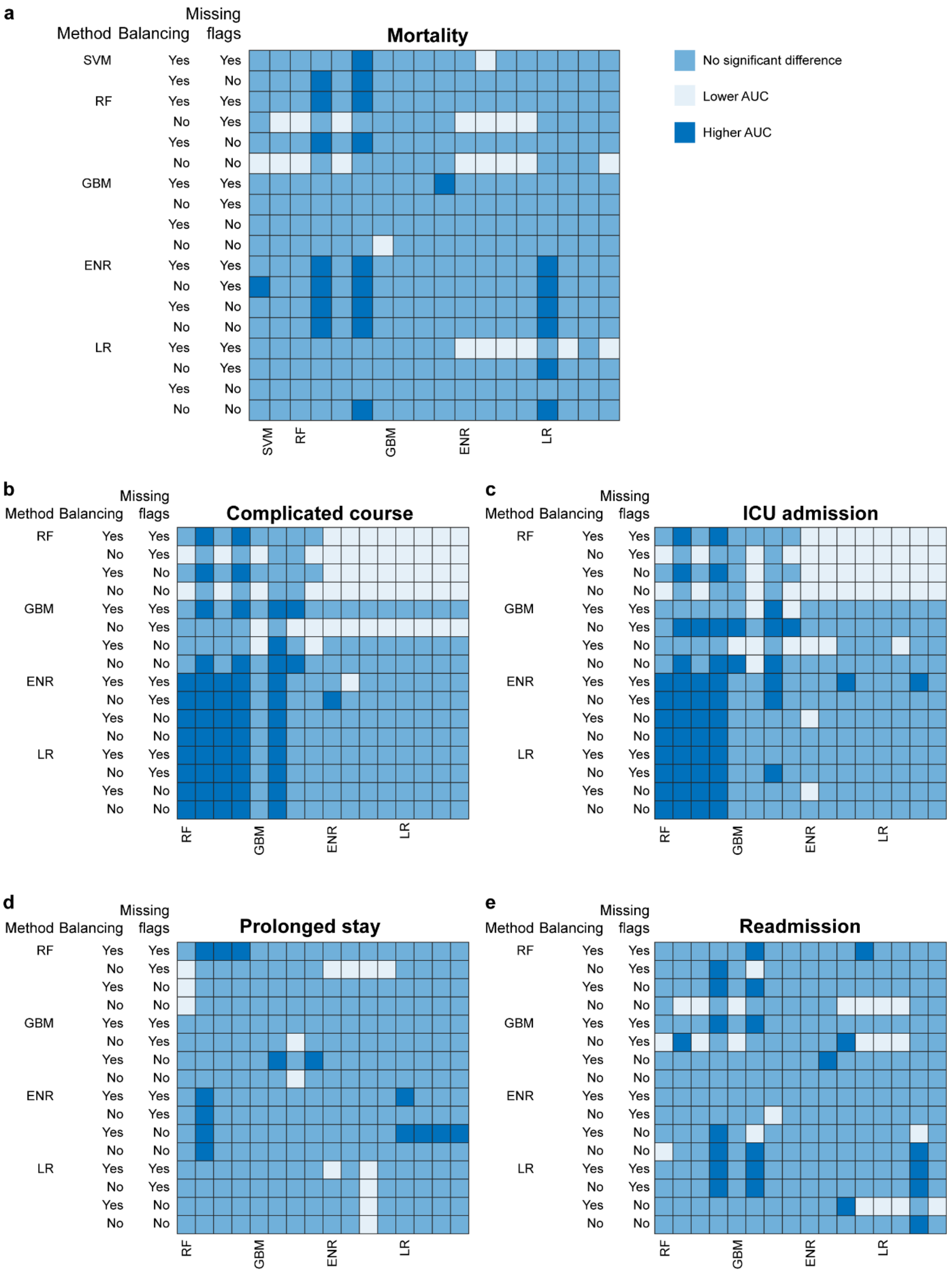


**eFigure 1:** Pairwise comparison of AUC scores for Elastic Net Regression, Random Forest, Gradient Boosting Models and Support Vector Machine for predicting mortality (**a**), complicated course(**b**), ICU admission(**c**), prolonged stay(**d**) and readmission(**e**). Models were compared by the deLong test, and significant differences (*P*<.05) were reported along with whether the model on the y-axis has a higher (dark blue) or lower (gray) AUC than the model on the x-axis. Models are ordered the same on both axis.

© 2021 van den Bosch T et al. *JAMA Network Open.*

**eFigure 2.** Significant Predictors in Multivariate Logistic Regression Models for Complicated Course

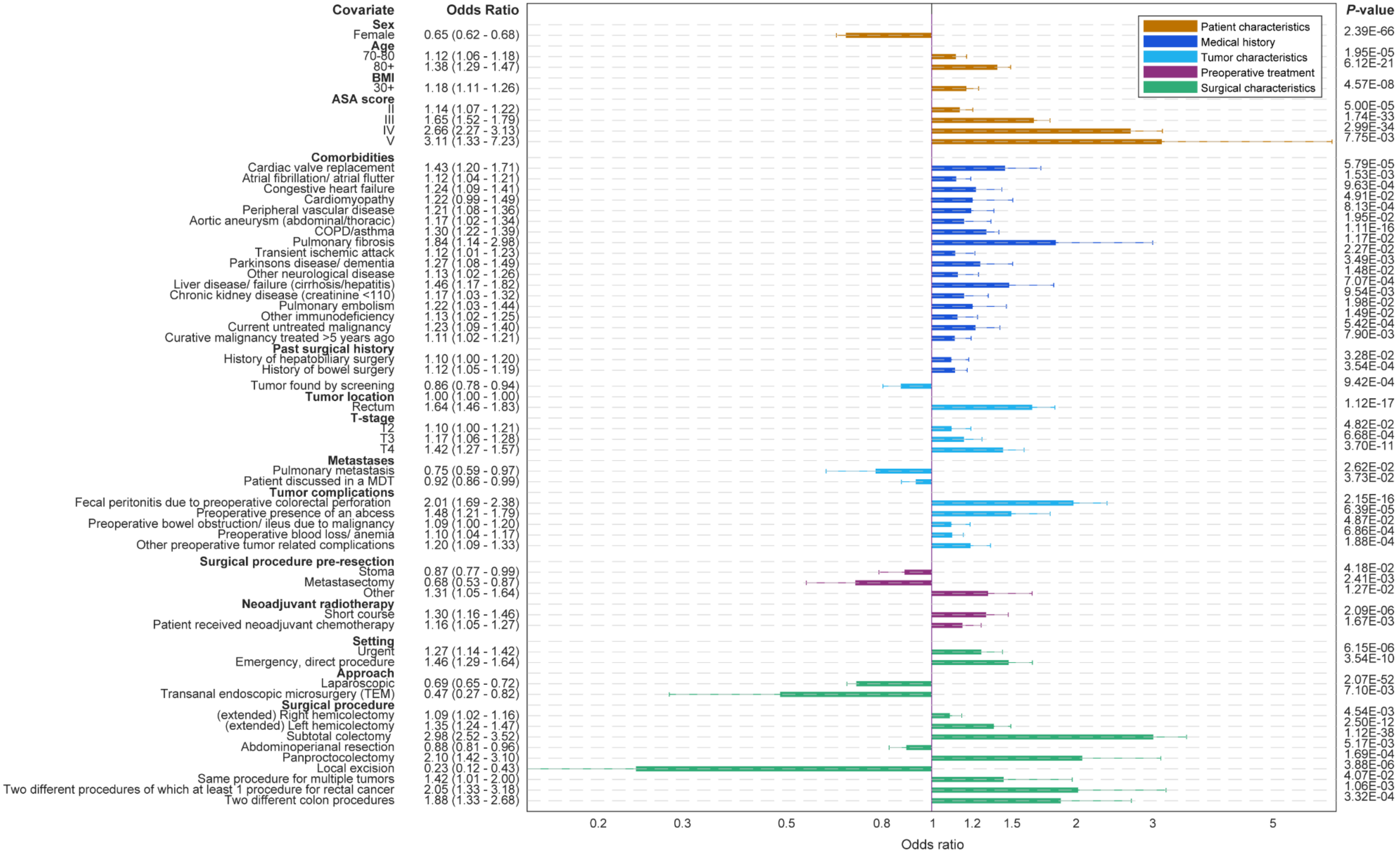


**eFigure 2:** Significant predictors in multivariate logistic regression model for complicated course on 62,501 patient records. All Regression coefficients with Wald *P*-value < 0.05 are translated to odds ratios and shown in the figure. Error bars represent 95% CIs. Variables are grouped by colors: Patient characteristics (red), comorbidities (blue), tumor characteristics (light blue), surgical complifcations (purple) and surgical characteristics (green). References values for categorical variables are Male (gender), 18.5-25 (BMI), 60-70 (age), ASA score I (ASA score), No pre-existing stoma (presence of a stoma before treatment), 1 tumor found (Number of colorectal tumors found by scopy), T1 (T-stage), M1 (M-stage), Colon Tumor (Tumor location), Elective (Setting), Open (Approach), low Anterior-resection/sigmoid resection (Surgical procedure), No conversion (Surgical convesion), No stoma (Stoma) and No intraoperative therapy (Radiotherapy of chemotherapy during surgery).

© 2021 van den Bosch T et al. *JAMA Network Open*.

**eFigure 3.** Significant Predictors in Multivariate Logistic Regression Models for ICU Admission

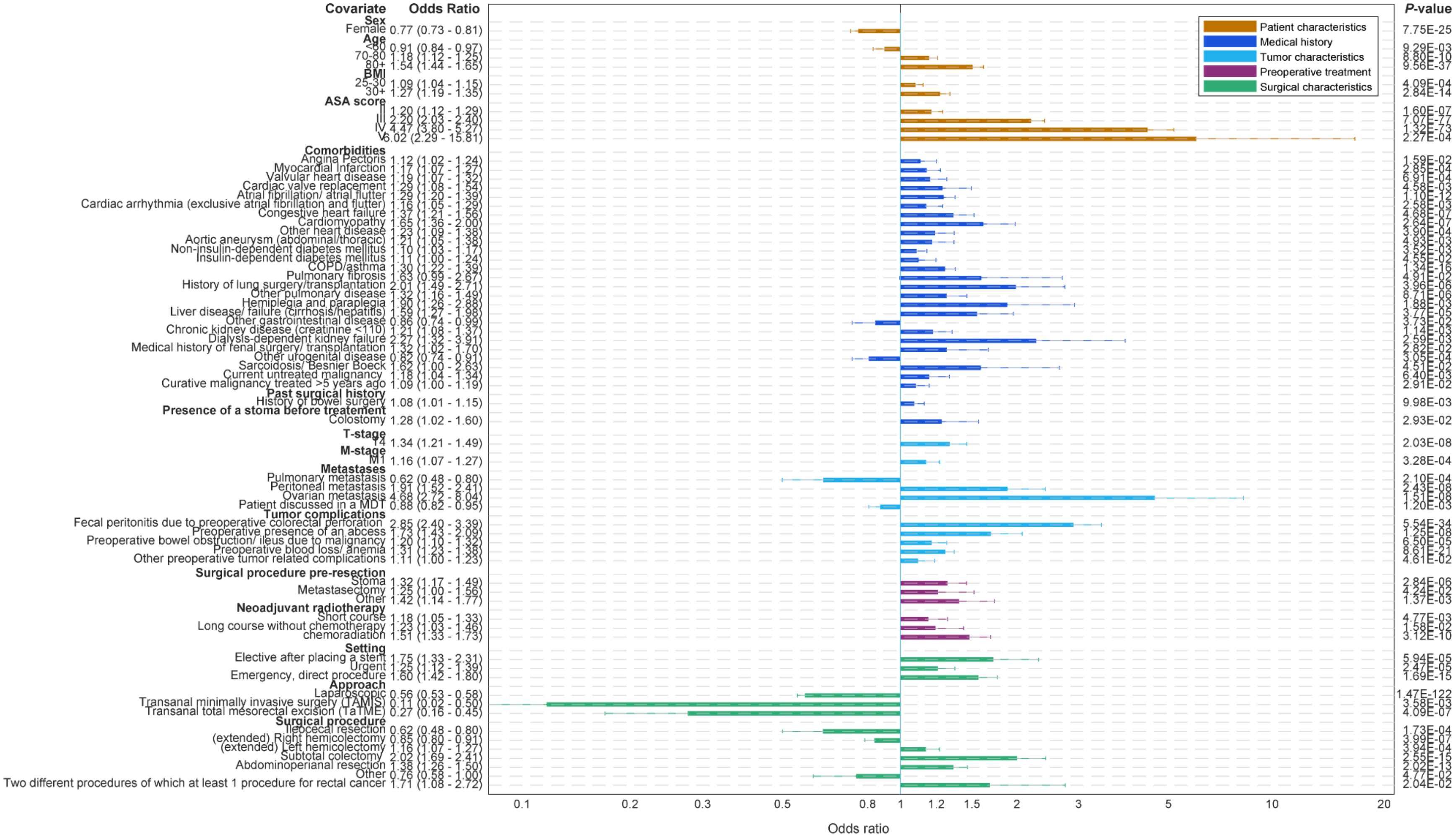


**eFigure 3:** Significant predictors in multivariate logistic regression model for ICU admission on 62,501 patient records. All Regression coefficients with Wald *P*-value < 0.05 are translated to odds ratios and shown in the figure. Error bars represent 95% CIs. Variables are grouped by colors: Patient characteristics (red), comorbidities (blue), tumor characteristics (light blue), surgical complifcations (purple) and surgical characteristics (green). References values for categorical variables are Male (gender), 18.5-25 (BMI), 60-70 (age), ASA score I (ASA score), No pre-existing stoma (presence of a stoma before treatment), 1 tumor found (Number of colorectal tumors found by scopy), T1 (T-stage), M1 (M-stage), Colon Tumor (Tumor location), Elective (Setting), Open (Approach), low Anterior-resection/sigmoid resection (Surgical procedure), No conversion (Surgical convesion), No stoma (Stoma) and No intraoperative therapy (Radiotherapy of chemotherapy during surgery).

© 2021 van den Bosch T et al. *JAMA Network Open.*

**eFigure 4.** Significant Predictors in Multivariate Logistic Regression Models for Readmission

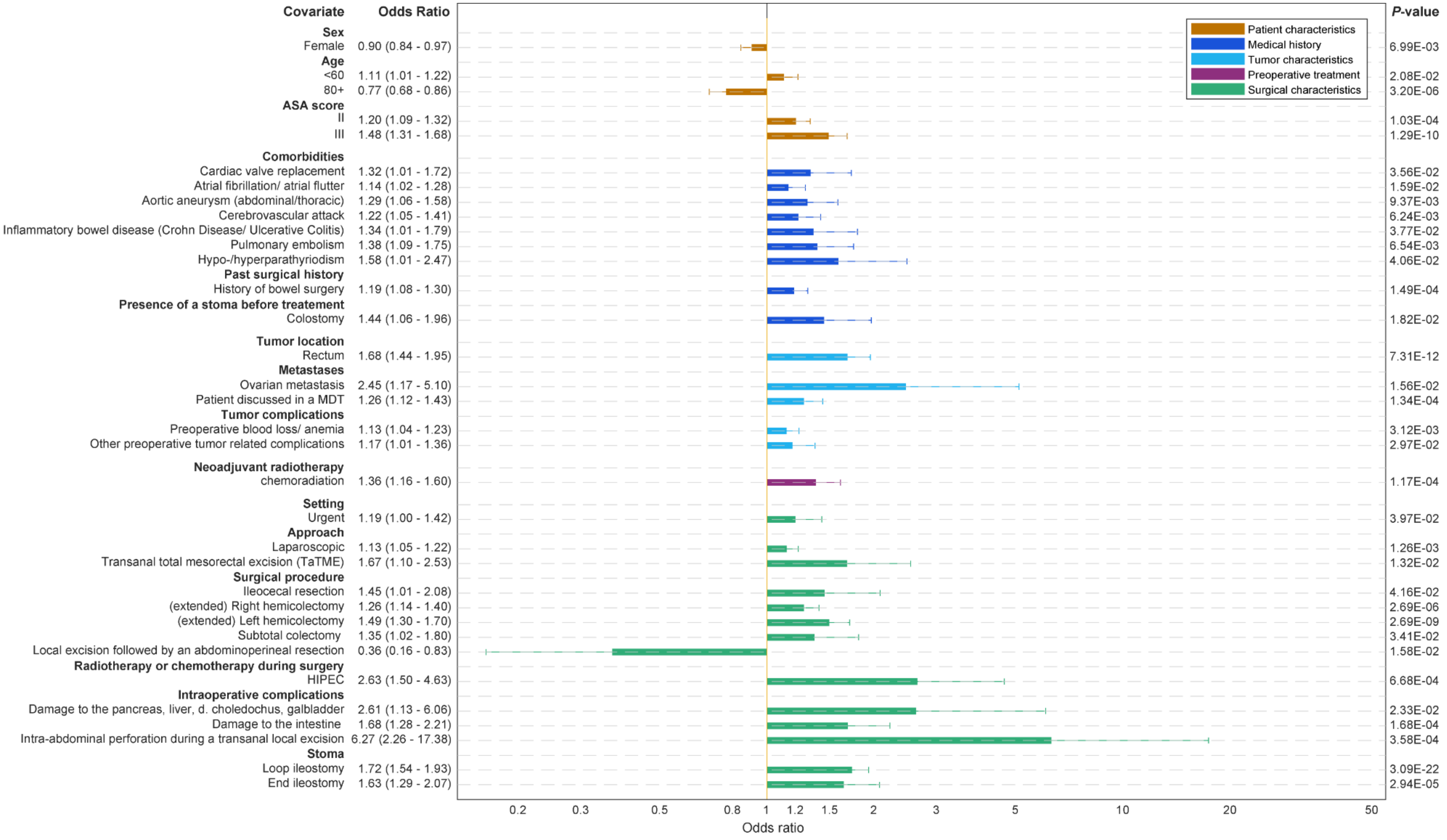


**eFigure 4:** Significant predictors in multivariate logistic regression model for readmission on 62,501 patient records. All Regression coefficients with Wald *P*-value < 0.05 are translated to odds ratios and shown in the figure. Error bars represent 95% CIs. Variables are grouped by colors: Patient characteristics (red), comorbidities (blue), tumor characteristics (light blue), surgical complifcations (purple) and surgical characteristics (green). References values for categorical variables are Male (gender), 18.5-25 (BMI), 60-70 (age), ASA score I (ASA score), No pre-existing stoma (presence of a stoma before treatment), 1 tumor found (Number of colorectal tumors found by scopy), T1 (T-stage), M1 (M-stage), Colon Tumor (Tumor location), Elective (Setting), Open (Approach), low Anterior-resection/sigmoid resection (Surgical procedure), No conversion (Surgical convesion), No stoma (Stoma) and No intraoperative therapy (Radiotherapy of chemotherapy during surgery).

© 2021 van den Bosch T et al. *JAMA Network Open.*

**eFigure 5.** Significant Predictors in Multivariate Logistic Regression Models for Prolonged Length of Hospital Stay

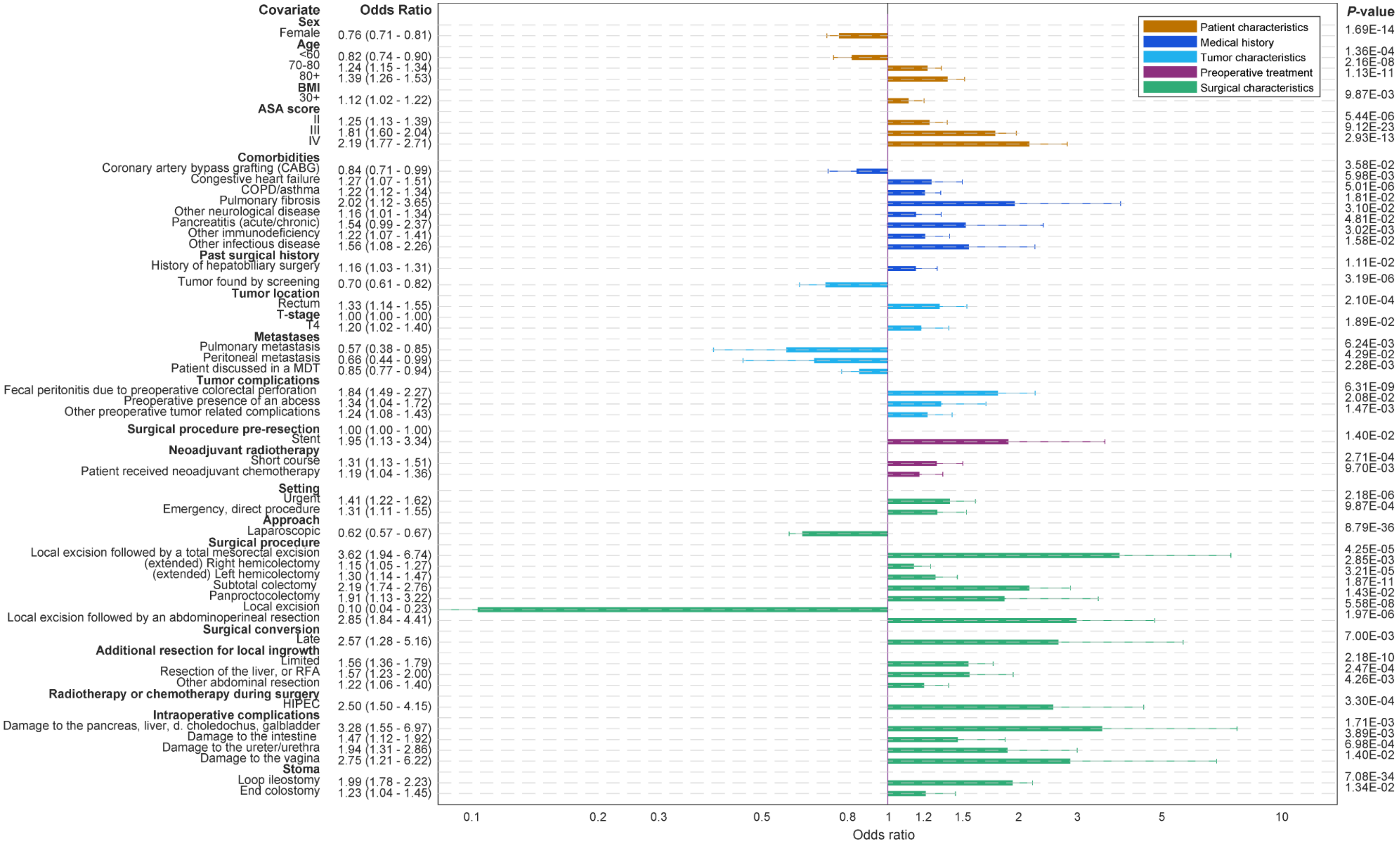


**eFigure 5:** Significant predictors in multivariate logistic regression model for prolonged length of hospital stay on 62,501 patient records. All Regression coefficients with Wald *P*-value < 0.05 are translated to odds ratios and shown in the figure. Error bars represent 95% CIs. Variables are grouped by colors: Patient characteristics (red), comorbidities (blue), tumor characteristics (light blue), surgical complifcations (purple) and surgical characteristics (green). References values for categorical variables are Male (gender), 18.5-25 (BMI), 60-70 (age), ASA score I (ASA score), No pre-existing stoma (presence of a stoma before treatment), 1 tumor found (Number of colorectal tumors found by scopy), T1 (T-stage), M1 (M-stage), Colon Tumor (Tumor location), Elective (Setting), Open (Approach), low Anterior-resection/sigmoid resection (Surgical procedure), No conversion (Surgical convesion), No stoma (Stoma) and No intraoperative therapy (Radiotherapy of chemotherapy during surgery).

© 2021 van den Bosch T et al. *JAMA Network Open.*

**eFigure 6.** Most Influential Predictor Variables for Complicated Course

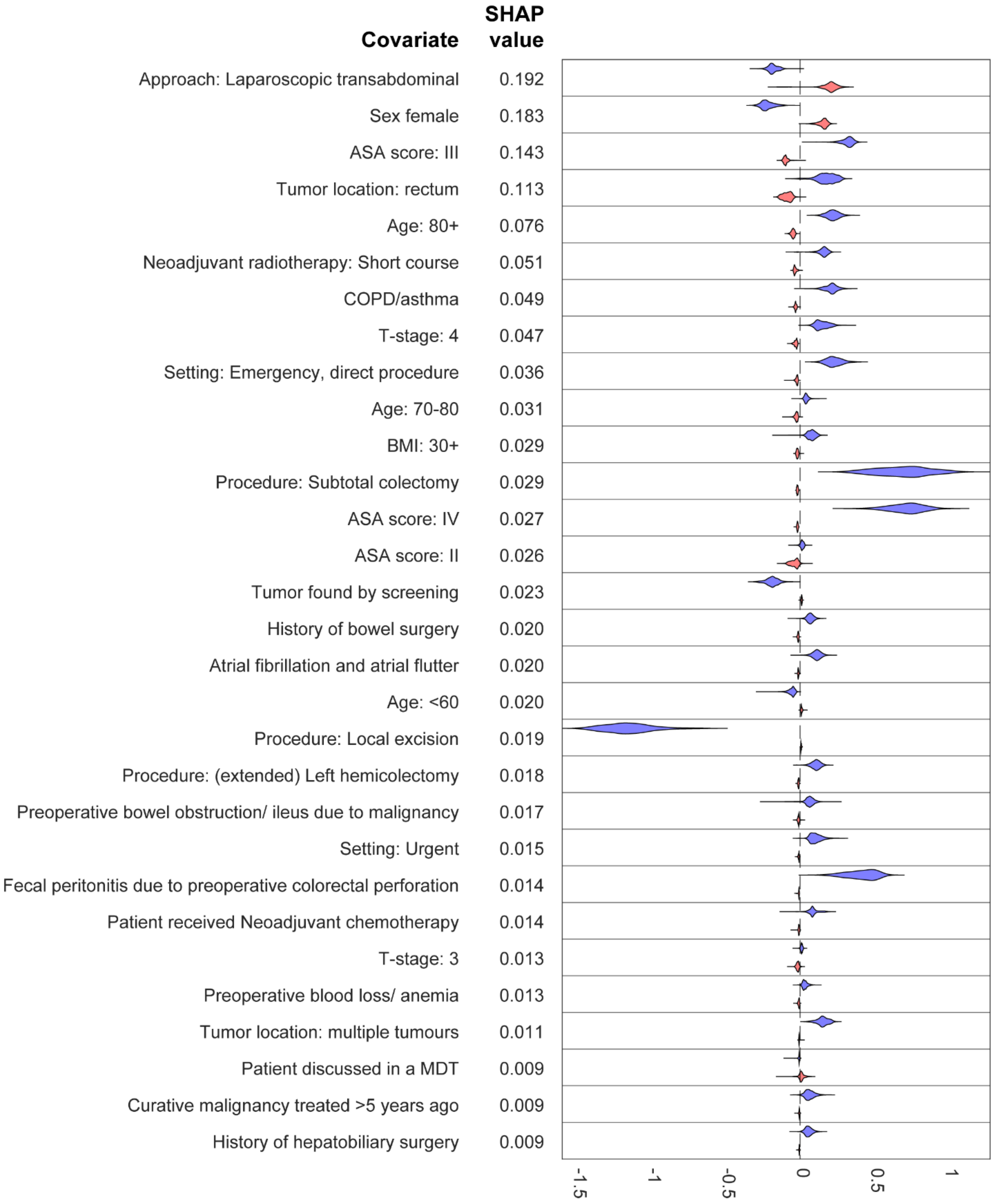


**eFigure 6:** Top 30 SHAP feature values of the gradient-boosting model for prediction of complicated course. Top 30 SHAP feature values of the gradient-boosting model for prediction of 30-day mortality. SHAP values were calculated per variable for all patients in the test set. Distributions of SHAP values for patients are shown in blue (patients that are positive for a variable) and red (patients that are negative for a variable). SHAP values were ranked by the mean of the absolute value across all patients in the test set.

© 2021 van den Bosch T et al. *JAMA Network Open.*

## **eFigure 7.** Most Influential Predictor Variables for ICU Admission

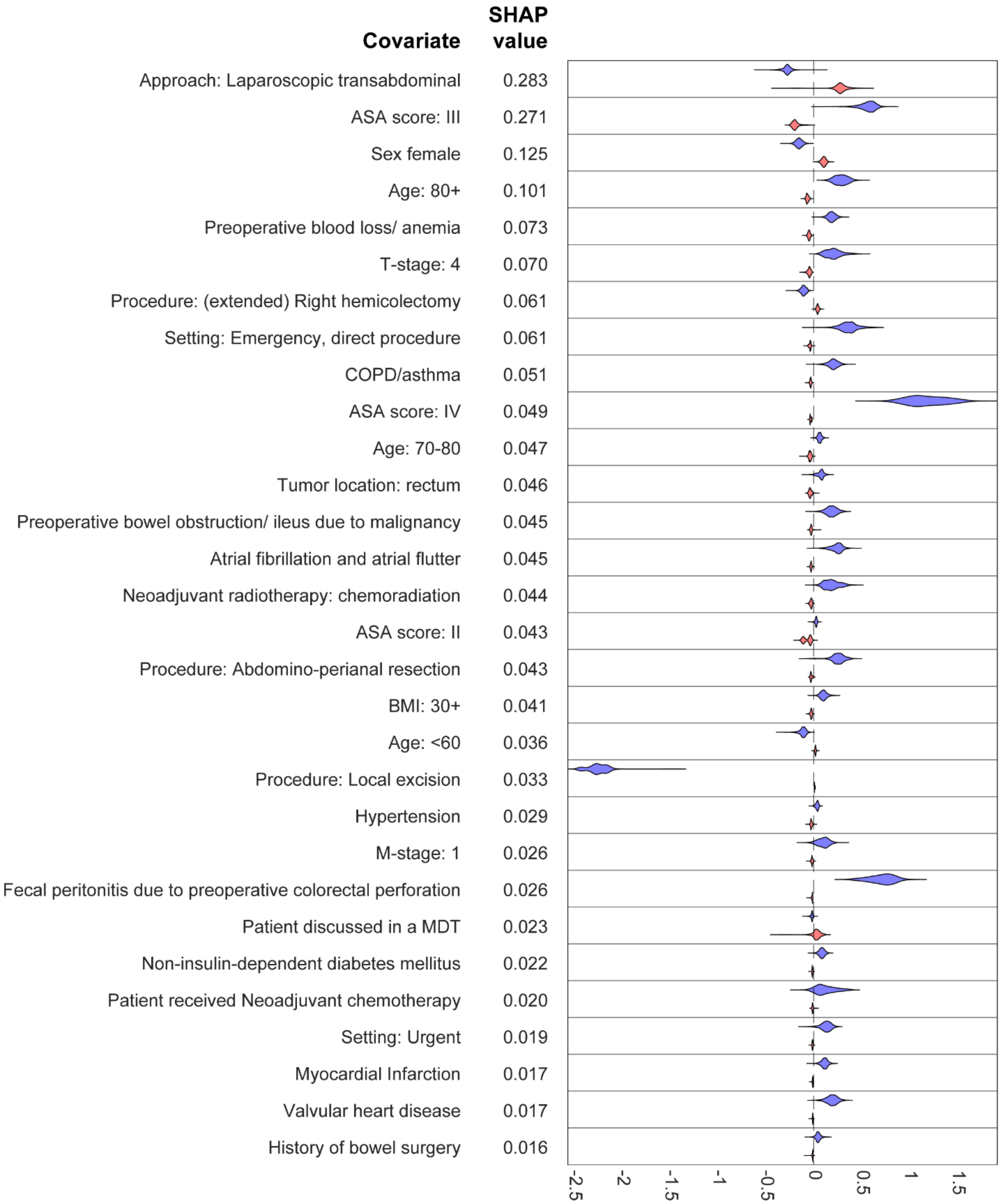


**eFigure 7:** Top 30 SHAP feature values of the gradient-boosting model for prediction of 30-day mortality. SHAP values were calculated per variable for all patients in the test set. Distributions of SHAP values for patients are shown in blue (patients that are positive for a variable) and red (patients that are negative for a variable). SHAP values were ranked by the mean of the absolute value across all patients in the test set.

© 2021 van den Bosch T et al. *JAMA Network Open.*

## **eFigure 8.** Most Influential Predictor Variables for Hospital Readmission

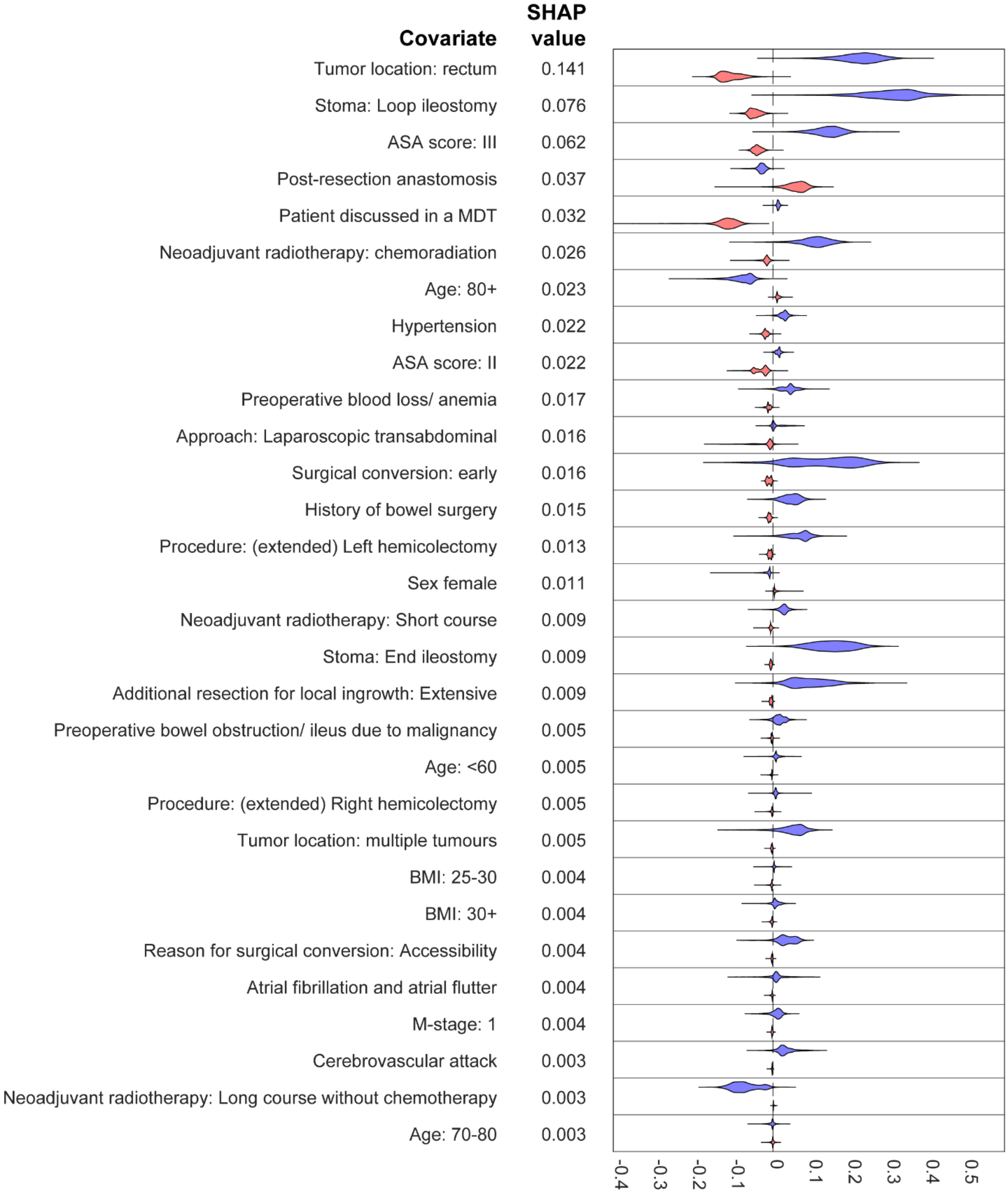


**eFigure 8:** Top 30 SHAP feature values of the gradient-boosting model for prediction of readmission. Top 30 SHAP feature values of the gradient-boosting model for prediction of 30-day mortality. SHAP values were calculated per variable for all patients in the test set. Distributions of SHAP values for patients are shown in blue (patients that are positive for a variable) and red (patients that are negative for a variable). SHAP values were ranked by the mean of the absolute value across all patients in the test set.

© 2021 van den Bosch T et al. *JAMA Network Open.*

**eFigure 9.** Most Influential Predictor Variables for Prolonged Length of Hospital Stay

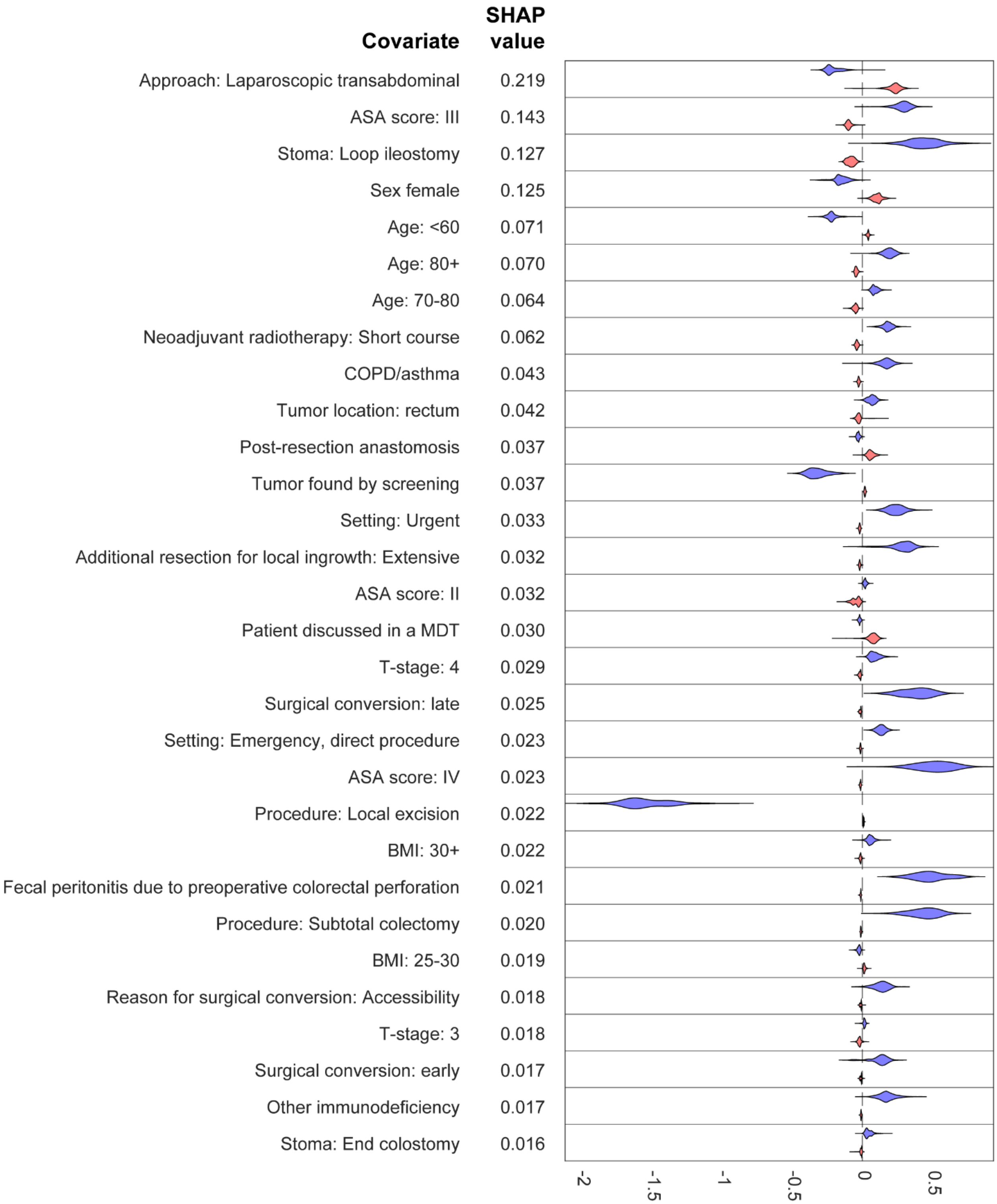


**eFigure 9:** Top 30 SHAP feature values of the gradient-boosting model for prediction of prolonged length of hospital stay. Top 30 SHAP feature values of the gradient-boosting model for prediction of 30-day mortality. SHAP values were calculated per variable for all patients in the test set. Distributions of SHAP values for patients are shown in blue (patients that are positive for a variable) and red (patients that are negative for a variable). SHAP values were ranked by the mean of the absolute value across all patients in the test set.

© 2021 van den Bosch T et al. *JAMA Network Open.*
